# Supplementary material for: Feasibility and metabolic outcomes of a well-formulated ketogenic diet as an adjuvant therapeutic intervention for women with stage IV metastatic breast cancer: The Keto-CARE trial
Source: PLoS One. 2024 Jan 2;19(1):e0296523. doi: 10.1371/journal.pone.0296523 (PMC10760925; doi:10.1371/journal.pone.0296523)
Supplement: S1 Table — (DOCX) [file pone.0296523.s001.docx]

| **Supplemental Table 1 \|** Drug Prescription | | | | |  |
| --- | --- | --- | --- | --- | --- |
| **Chemical Name** | **Trade Name** | **Administration Method** | **Primary Indication** | **Method of Action** |  |
| Paclitaxel | Taxol | Injection | Anthracycline resistance | Antimicrotubule agent |  |
| Capecitabine | Xeloda | Pill | Anthracycline resistance | Neoplasm inhibitor |  |
| Doxorubicin | Doxil | Injection | Ovarian metastasis | Topoisomerase inhibitor |  |
| Palbociclib | Ibrance | Pill | Identified HER2- | CDK inhibitor |  |
| Exemestane | Aromasin | Pill | Postmenopausal women | Aromatase inhibitor |  |
| Letrozole | Femara | Pill | Postmenopausal women | Aromatase inhibitor |  |
| Denosumab | Xgeva | Injection | Bone metastases | RANK inhibitor |  |
| Goserelin | Zoladex | Subcutaneous Implant | Estrogen deprivation | GnRH analogue inhibitor |  |
| Peruzumab | Perjeta | Injection | EGF inhibition; HER2+ | MAP/PI3K inhibitor |  |
| Trastuzumab | Herceptin | Injection | EGF inhibition; HER2+ | ADCC mediator |  |
| Docetaxel | Taxotere | Injection | Tubulin stabilization; VEGF | Mitotic/Neoplasm inhibitor |  |
| *ADCC: antibody dependent cellular toxicity; CDK: cyclic dependent kinase; EGF = epidermal growth factor; HER2-/+ = Herceptin node negative/positive; MAP: mitogen activated protein; PI3K: phosphoinositol-3-kinase; RANK: receptor activator of NF-κB.* | | | | |  |
|  |  |  |  |  |  |
