# Supplementary material for: Feasibility and metabolic outcomes of a well-formulated ketogenic diet as an adjuvant therapeutic intervention for women with stage IV metastatic breast cancer: The Keto-CARE trial
Source: PLoS One. 2024 Jan 2;19(1):e0296523. doi: 10.1371/journal.pone.0296523 (PMC10760925; doi:10.1371/journal.pone.0296523)
Supplement: S2 Table — (DOCX) [file pone.0296523.s002.docx]

| **Supplemental Table 2 \|** Menu Items |
| --- |
| **Vegetables and Fruits** |
| *Leafy Greens* |
| Cruciferous vegetables (kale, collard greens, watercress, broccoli, brussels sprouts, kohlrabi, cauliflower, bok choy, rapini, (broccoli rabe), cabbage, arugula, radish) |
| Lettuces (especially romaine) |
| Parsley |
| Spinach |
| *Other Common Vegetables* |
| Artichoke |
| Asparagus |
| Avocados |
| Celery |
| Cucumber |
| Eggplant |
| Endive |
| Mushrooms |
| Onions |
| Peppers |
| Radicchio |
| Snow peas |
| Summer squash |
| Tomatoes |
| Wax beans (green and yellow) |
| Zucchini |
|  |
| **Fruits** |
| Lemons and limes (used sparingly for flavoring teas and water) |
| Olives |
| Berries (blackberries, raspberries, strawberries, blueberries) |
| Apples (sparingly) |
| Kiwi (sparingly) |
| Pears (sparingly) |
|  |
| **Proteins** |
| Eggs |
| Fish (salmon, tuna, sardines) |
| Shellfish |
| Poultry (chicken, turkey, duck, goose) |
| Pork (bacon, ham, prosciutto, or serrano) |
| Beef |
| Lamb |
| Sausage |
| Tofu, tempeh, and textured vegetable protein (vegetarian option) |
| Non-wheat, non-grain flours* (almond, coconut, soy) |
|  |
| **Dairy** |
| Cheese |
| Yogurt (plain, unsweetened) |
| Whipping cream |
| Full-fat sour cream |
| Full-fat cream cheese |
| Kefir (unsweetened) |
|  |
| **Fats and Oils** |
| Butter |
| Olive oil |
| Macadamia oil |
| Avocado oil |
| Coconut oil |
| Beef tallow |
|  |
| **Nuts and Seeds** |
| Chia seeds |
| Flax seeds |
| Hemp seeds |
| Pumpkin seeds |
| Almonds (including almond flour and almond butter) |
| Brazil nuts |
| Hazelnuts |
| Macadamia nuts |
| Pecans |
| Pistachios |
| Walnuts |
|  |
| **Flavorings** |
| Salt (iodized) |
| Herbs and spices |
| Basil |
| Black pepper |
| Cardamom |
| Cayenne pepper |
| Chives |
| Cilantro |
| Cinnamon |
| Cloves |
| Garlic |
| Ginger |
| Nutmeg |
| Oregano |
| Parsley |
| Sage |
| Rosemary |
| Turmeric |
| Vinegar |
| Mustards (unsweetened) |
| Hot sauces (unsweetened) |
| Soy sauce |
| Extracts (vanilla, almond, maple) |
| Sugar substitutes (e.g., xylitol or erythritol, used sparingly) |
|  |
| **Miscellaneous** |
| Olives |
| Pickles (made with no sugar) |
| Pork rinds (chicharrons) |
| Beef jerky |
| Sugar-free chocolate (small amounts, 1 oz/day maximum) |
| Sugar-free candies (small amounts, 1–2/day maximum) |
