# Supplementary material for: Feasibility and metabolic outcomes of a well-formulated ketogenic diet as an adjuvant therapeutic intervention for women with stage IV metastatic breast cancer: The Keto-CARE trial
Source: PLoS One. 2024 Jan 2;19(1):e0296523. doi: 10.1371/journal.pone.0296523 (PMC10760925; doi:10.1371/journal.pone.0296523)
Supplement: S1 File — (DOCX) [file pone.0296523.s003.docx]

## Clinical Protocol

**KETOgenic Diet and Chemotherapy to Affect REcurrence of Breast Cancer (The KETO-CARE Study)**

### 1. METASTATIC BREAST CANCER

Deaths attributed to breast cancer in the US exceed 40,000 annually, the majority of which are due to metastatic disease (American Cancer Society, 2015). Outside of standard radiological and pharmacological treatments, there are few evidence-based options for patients with metastatic disease. Although there is great interest in natural, homeopathic and nutritional strategies, the vast majority of National Comprehensive Cancer Networks do not provide dietary recommendations, and the ones that do are inconsistent (Champ et al., 2013). Given the strong connection between specific dietary patterns and metabolic/physiologic processes known to be associated with breast cancer (e.g., body fat, inflammation, oxidative stress, insulin resistance, metabolic syndrome), there is a clear need to study nutrition-based metabolic therapies that may work in synergy with chemotherapy treatment. ***To date there is no known cure for metastatic breast cancer, but case studies show that adherence to a ketogenic diet can result in full remission of metastases*** (Jansen and Walach, 2016). Given the robust metabolic adaptations associated with well formulated ketogenic diets and recently identified non-metabolic roles of ketones that target the complex pathology of cancer, a more rigorous clinical study is warranted to investigate the therapeutic applications of a diet that induces nutritional ketosis in women with metastatic breast cancer.

### 2.0 STANDARD OF CARE (PACLITAXEL)

Paclitaxel (Taxol) is currently utilized as the initial palliative chemotherapeutic agent at the Stefanie Spielman Comprehensive Breast Cancer Center at The Ohio State University as well as in other centers throughout the US due to results of multiple studies showing improved disease free survival and overall (Ghersi et al., 2015; Giorgio Mustacchi, 2015). Paclitaxel is a weekly regimen associated with a median progression free survival of approximately 12 months, although some cases of TNBC behave aggressively with very short disease free intervals. Paclitaxel has two primary mechanistic actions via which it mediates the reduction in cancer proliferation: 1) cell cycle arrest and 2) induction of apoptosis. Taxol inhibits the disassembly of microtubules locking the microtubule in a rigid, polymerized state (Cang et al., 2014). As a result, the cell is blocked from advancing through mitosis and therefore cannot proliferate. Taxol also inhibits Bcl-2, an anti- apoptotic protein, and may induce apoptosis (Cang et al., 2014). However, similar to most other chemotherapeutic agents, patients are unable to undergo rigorous dosing protocols or long-term infusions due to chemotoxicity and associated side effects (e.g., neuropathy) (Liu et al., 2013). Due to hypersensitivity reactions to the solvent of paclitaxel, premedication with steroids is the standard of care.

### A NOVEL METABOLIC THERAPY FOR COMBINATORY CARE WITH FIRST-LINE CHEMOTHERAPY

Most of the knowledge regarding diet and breast cancer is from association studies and does not provide any clear guidance as to the best anti-cancer eating pattern. Very little

intervention work has focused on MBC, but in earlier stages of breast cancer most studies focused on diets low in fat and high in carbohydrate with mixed results. The WHEL study, Women’s Health Initiative (WHI) and Women's Intervention Nutrition Study (WINS) all failed to demonstrate that a low fat, plant based diet decreased the likelihood of cancer recurrence or treatment (Blackburn and Wang, 2007; Pierce et al., 2002; Prentice et al., 2006). The WINS study did in fact demonstrate improvements in BC occurrence; this however was a result of weight loss, and not attributable to dietary intake (Blackburn and Wang, 2007). Since women with MBC can live for several years after initial diagnosis and do not initially suffer from cancer cachexia, as in other metastatic disease types, targeted dietary interventions early in the disease course may confer substantial benefits.

The unique metabolism and progression pathways of advanced stage BC (i.e. insulin- dependent growth mechanisms, body weight-based prognosis etc.) provide a unique opportunity to improve treatment and quality of life with a combinatory treatment of nutrition and chemotherapy. We believe the metabolic state of nutritional ketosis induced by a very low-carbohydrate diet has the potential to improve survival and quality of life in women with MBC.

### Metabolic Adaptations to a Ketogenic Diet and Relevance to Breast Cancer

A ketogenic diet causes accelerated fat metabolism, which results in production of specific metabolites called ketones. The principle ketone body beta-hydroxybutyrate (BOHB) is being studied in much greater detail as both a metabolite and potent signaling molecule capable of acting like a hormone (Newman and Verdin, 2014a; 2014b). Over the last 15 years, our research group has established the superiority of very low-carbohydrate ketogenic diets over traditional low-fat diets in managing insulin resistant conditions (Feinman et al., 2015; Volek et al., 2008), which now includes over half the adults in the US (Menke et al., 2015). The metabolic state of nutritional ketosis is associated with a robust shift to almost exclusive reliance on fatty acids and ketones for fuel. Interestingly, highly competitive national caliber ultra-endurance athletes are also increasingly switching to ketogenic diets. We recently published the first paper showing that keto-adapted athletes have extraordinary fat burning capabilities at least 50% higher than the highest rates ever recorded (Volek et al., 2015). The reduced reliance on carbohydrate oxidation and insulin- mediated glucose uptake has beneficial effects on satiety, weight loss, insulin sensitivity and glycemic control. There is also decreased inflammation and oxidative stress, improvements in cholesterol and lipoprotein profile, fatty acid composition, and overall cardio-metabolic risk. Many women in the early phases of MBC are overweight, prediabetic, or diabetic – all of which contribute to higher mortality and poorer responses to cancer treatment. Thus, women with MBC would benefit from a ketogenic diet in managing these comorbidities.

Importantly, the state of nutritional ketosis should provide a favorable environment to nourish the body while deterring tumor growth. One central tenant of tumor metabolism is an almost exclusive uptake and utilization of glucose for energy derivation. An ability to control blood glucose and insulin, without additional medication, may inherently decrease tumor viability. The reliance on glucose as a fuel substrate is exploited via 18-

flourodeoxyglucose positron emission tomography (FDG-PET), the gold standard for cancer diagnosis and prognostic characterization. Evidence that targeting insulin sensitivity and improved glucose management is benefical to cancer comes from the recent discovery that metformin, the most widely used anti-diabetic medication to improve insulin sensitivity, results in decreased risk of cancer occurrence (Sahra, 2010). Nutritional ketosis resulting from a very low-carbohydrate ‘ketogenic’ diet consistently reduces circulating plasma glucose levels and increases insulin sensitivity (Boden et al., 2005; Dashti et al., 2006; Gumbiner et al., 1996).

Given the evidence that ketogenic diets improve insulin sensitivity, which in turn appears to improve risk for cancer, In fact, the two small clinical trials that investigated the effectiveness of a ketogenic diet in advanced stage breast cancer patients both demonstrated slowed disease or partial remission in several patients (Fine et al., 2012; Schmidt et al., 2011). A recent study reported that a patient with breast cancer with PET avid metastasis in both lung and bone experienced complete remission after adoption of a ketogenic diet (Jansen and Walach, 2016). The same authors further demonstrated that 60% of the adopters experienced improvements in tumor biology or prognosis, with diet adherence associated with better outcomes (Jansen and Walach, 2016). ***These findings provide a strong scientific rationale for a larger and tightly controlled study in advanced cancer patients that incorporates more sophisticated methods of inducing and monitoring nutritional ketosis.***

It is well understood that cancer results in a state of chronic inflammation, in which the tumor thrives. Ketogenic diets have clinically demonstrated improved systemic inflammation status, specifically decreased concentrations of several proinflammatory cytokines (e.g., IL-6 and IL-8) (Forsythe et al., 2008), both of which are significantly increased in advanced stage breast cancer patients (Kozłowski et al., 2003). This may significantly improve patient well-being and quality of life, as cancer related fatigue in BC patients has been linked with chronically elevated concentrations of the pro-inflammatory cytokine IL-6 (Bower, 2007). IL-6 increases have also been linked with an induction of the genes SNAIL and TWIST. SNAIL and TWIST are two of the major genetic regulators of epithelial-mesenchymal transition (EMT), the initial step of metastasis (Poillet-Perez et al., 2015). Nutritional ketosis has been found to reduce the amount of cancer stem cells, a cell population that is theorized to be the root cause for metastasis and cancer recurrence (Martuscello et al., 2015). Further *in vivo* evidence has demonstrated the ability of a ketogenic diet to improve mood of individuals undertaking a weight loss program (McClernon et al., 2007). *In vitro* research has demonstrated the ability of a ketogenic diet to decrease tumor viability and improve survival time (Poff et al., 2015).

One potential pathway for the discord in energy metabolism of BC may result from PTEN and phosphoinositide 3-kinase (PI3K) pathway mutations. Within the past two decades the tumor suppressor capabilities of the phosphatase and tensin homologue (PTEN) gene have been well documented (Song et al., 2012). Either PTEN inhibition or PI3K activation via insulin-mediated processes causes metabolic deregulation and result in a Warburg-like Effect. Loss of PTEN results in phosphatidylinositol-3,4,5-triphosphate dephosphorylation, as well as an increased phosphorylation of AKT via PDK1 further activating and enhancing

the PI3K/AKT pathway. Since insulin is a primary activator of the PI3/AKT pathway, tumor growth could reasonably be inhibited by therapeutic interventions that lower insulin concentration and action. One of the fundamental adaptations associated with keto- adaptation is decreased insulin concentration and signaling, increased reliance on fatty acids and ketones as energy substrates, and dramatically reduced reliance on glucose uptake and oxidation (Phinney et al., 1983; Volek et al., 2015). These lipid-based fuels do not require insulin-depending PI3K signaling for transport and oxidization in cells, and thus should, result in decreased action of PI3K and downstream effectors including mTOR inhibition (Martuscello et al., 2015).

Aside from direct effects on tumor based outcomes, nutritional ketosis may counteract undesirable side effects associated with drugs commonly used to manage BC. In efforts to combat inflammation associated with chemotherapeutic agents, patients are frequently administered glucocorticoids. Glucocorticoids function by binding the glucocorticoid receptor, activating transcription factors, and effecting target genes (Ferris & Kahn, 2012 JCI). While high doses glucocorticoids strengthen anti-inflammatory defenses and combat adverse reactions to chemotherapeutic drug infusion, they promote insulin resistance and weight gain (Ferris & Kahn, 2012 JCI). In fact, approximately two-thirds of individuals with a high dose of glucocorticoids exhibit hyperglycemic conditions (Donihi et al, 2006 Endocr Pract). Glucocorticoid-induced insulin resistance manifests in a similar fashion to that of Type II diabetes. The resultant hyperglycemia is associated with increased proteolysis, *de novo* lipogenesis, and hepatic fatty acid accumulation. These unfavorable metabolic outcomes to glucocorticoids are targeted by ketogenic diets (Feinman et al., 2015; Volek et al 2008). Thus, nutritional ketosis would be expected to decrease the need for glucocorticoids owing to its anti-inflammatory effects. In more aggressive cases, nutritional ketosis may also permit the use of higher doses of glucocorticoids, when needed, by mitigating untoward side effects.

Additionally, dexamethasone (the most common glucocorticoid prescribed with paclitaxel) has been demonstrated to minimize the characteristic decreased appetite and weight loss that occurs with chemotherapeutic drug infusion (Sarcev et al., 2008 Med Pregl). We expect that a ketogenic diet in combination with dexamethasone will serve as a force multiplier and further reduce weight loss.

Thus, there are several metabolic adaptations to a ketogenic diet that should benefit women with MBC including decreased fat mass, decreased glucose flux into tumors, less insulin burden, less inflammation and oxidative stress, improved tolerance to chemotherapy, and mitigation of side effects to medications.

### Non-Metabolic Roles of Ketones and Relevance to Breast Cancer

A defining feature of a well-formulated ketogenic diet is that circulating levels of BOHB increase by an order of magnitude. A remarkable new perspective on BOHB was published in Science just a few years ago (Shimazu et al., 2013a). This paper showed that BOHB is a potent histone deactylase (HDAC) inhibitor and regulator of a group of genes that protect cells from oxidative stress. Specifically, it was demonstrated that at physiological levels

characteristic of nutritional ketosis, BOHB switched on specific genes that protect cells from free radical damage. Shortly after this research was published, others reported that the same mechanism of action by BOHB also potently and directly reduced insulin resistance. Since oxidative stress is prominent in the pathophysiology of aging, it has been hypothesized that BOHB may be a longevity metabolite, which is now supported by two recent papers (Newman and Verdin, 2014b; 2014c).

It is highly probable that the nontoxic, epigenetic, drug-like effect of naturally-produced BOHB on HDAC inhibition has relevance to cancer metabolism and management. HDAC inhibitors are currently used as novel anti-cancer agents, and have been shown to arrest tumor growth and induce apoptosis in cancer cells in vitro and in vivo (Liu et al., 2013). Paclitaxel (taxol) is used as a first line standard of care chemotherapeutic drug for women with MBC. Two recent papers (Cang et al., 2014; Liu et al., 2013) demonstrated a potent synergistic effect of combining paclitaxel with an HDAC inhibitor on two different breast cancer cell lines. The HDAC inhibitor used in these studies was phenethyl isothiocyanate (PEITC), found in a wide variety of cruciferous vegetables with much less relative potency compared to BOHB. Further evidence demonstrating a therapeutic role of HDAC inhibitors has been the clinical success of Vorinostat in combating triple negative breast cancer and sensitizing the malignancy to more common treatments (Ha et al., 2014 Oncotarget). Vorinostat is a broad spectrum HDAC inhibitor that selectively targets similar HDACs as BOHB (Ha et al., 2014; Shimazu et al., 2013). Thus it may be likely that a ketogenic diet provides a non-toxic pathway for improving patient-related outcomes and increase tumor sensitivity to the taxane therapy. In our proposed study, we will customize the diet such that we optimize ketones in the same range shown to result in HDAC inhibition (Shimazu et al., 2013a). We expect this unique synergistic combination will yield overall favorable patient effects (e.g., less tumor growth, decreased treatment dose to maintain effectiveness, decreased toxicity of treatment).

### Current Knowledge of Ketogenic Diets in Cancer

There currently exist several basic science papers and animal studies pointing to positive effects of ketogenic diets in different types of cancers (Poff et al., 2015; 2014; Stafford et al., 2010), and this is now moving into human clinical trials. Two previous studies have found a high degree of feasibility of the ketogenic diet in human patients with advanced stage BC (Fine et al., 2012; Schmidt et al., 2011). Both research teams successfully demonstrated that a ketogenic diet is feasible and well tolerated with no adverse events in advanced stage cancer patients. In a cohort analysis Fine (2012) demonstrated that participants with the highest ketosis levels exhibited either stable disease, or partial remission. While findings were promising, the sample and effect sizes were small, the diets lacked sophistication and were at the lower end of nutritional ketosis, and they were not done in combination with chemotherapy. The patients studied in Fine (2012) were also later stage 4 and thus represented a population that may be more difficult to treat effectively than women initially diagnosed with MBC. A larger study with better control over dietary parameters is needed to determine the response to a ketogenic diet in women with MBC. Despite the sample size limitations, interpretation of the Fine (2012) study reveals that the highest responders to ketosis were most likely to succeed. This finding corroborates results from

Schmidt (2011) that showed participants who completed the intervention had higher levels of stable disease or partial remission. There currently exist approximately ten registered clinical trials investigating a ketogenic diet in cancer patients, the majority of which are focused on head, neck, lung and brain primary tumor sites. None of these trials are investigating MBC patients. Due to the unique etiology and most common tumorigenic pathways, MBC presents a myriad of potential therapeutic mechanisms through which ketosis may work.

Thus, the next logical step is to perform a larger study in women with MBC that includes a well-formulated ketogenic diet by investigators that appreciate the nuances in diet formulation and need for personalization. Our research group has two decades of experience studying ketogenic diets and is uniquely suited to perform such a study.

### RATIONALE AND FEASIBILITY

Currently, women diagnosed with Stage 4 MBC experience a significant mortality rate, with 20% survival greater than 5 years. We believe these statistics could be improved dramatically while dramatically improving quality of life with a highly palatable diet that induces nutritional ketosis. Two previous studies determined that a ketogenic diet is safe and feasible in a glucose-dependent breast cancer population. However, both the findings of Fine (2012) (Fine et al., 2012) and Schmidt (2011) suffered from very small sample sizes and the lack of a control group. Further, both of the aforementioned trials suffered from an inability to control diet, even in the intervention group. This resulted in variable and inconsistent ketone concentrations, as well as varied responses to treatment.

As mentioned previously, nutritional ketosis is a pleiotropic therapeutic approach to cancer treatment. A strong body of evidence has demonstrated the clinical benefits of a ketogenic diet in intractable epilepsy and conditions associated with insulin resistance and its various manifestations (e.g., obesity, metabolic syndrome, type-2 diabetes), which now includes cancer. Keto-adaptation results in less reliance on the glucose/insulin axis and profound changes in substrate use characterized by accelerated fatty acid oxidation and decreased glucose flux, which may provide a therapeutic mechanism for treatment of breast cancer. Keto-adaptation also restores the hormonal and inflammatory environment of the host in ways that would be expected to deter tumor growth. Decreased insulin concentration and signal transduction should translate into less activation of growth factors and oncogenic pathways associated with PI3K/Akt, mTOR and HIF. Keto-adaptation promotes an anti- inflammatory phenotype of the patient that may result in a decreased invasiveness and increased progression free survival.

Our research team has an established expertise in conducting ketogenic diet interventions. We propose to conduct a highly controlled feeding study for three months to examine the effects of sustained, individualized nutritional ketosis on tumor growth and biological/behavioral measures, followed by transition to a “free-living” period with coaching to demonstrate this unique metabolic state can be sustained for another 3 months with continued favorable outcomes.

### OBJECTIVES

Many individuals have adopted a low-carbohydrate diet for health reasons, yet there is scarce professional support available to provide guidance and support. We have scientific expertise and practical knowledge of ketogenic diets combined with a passion to empower people with the tools to implement this eating approach into their lifestyle. This project is highly patient-centered. We will support women who voluntarily choose to be in the intervention group and provide them with a personalized eating plan designed to have maximal therapeutic impact and positively impact their lives. To that end, this project is unique in that it is highly patient-centered while also designed to have a substantial scientific and practical impact on medical management of breast cancer. The main objectives are as follows:

### OBJECTIVE 1. To evaluate the feasibility of implementing a diet that induces nutritional ketosis in women who are initiating palliative chemotherapy to treat advanced stage BC.

1a. During the first 3-month period all meals will be prepared and provided to women to ensure a target therapeutic level of ketosis 1.5-5.0 mmol/L (typical levels are <0.2 mmol/L). Blood ketone concentrations will be measured daily and the diet individually adjusted if ketones are low (e.g., decrease the carbohydrate and/or protein).

2a. During the second 3-month period all women will transition to a ‘free-living’ ketogenic diet. We will employ a behavior-based educational coaching model that incorporates group, individualized, and digital content to participants.

As we have shown many times before in different populations ranging from diabetics to elite athletes, we hypothesize that women with metastatic BC will successfully accept both a controlled feeding and free-living ketogenic diet as evidenced by nutrient assessment, food satisfaction questionnaires, and blood ketone measurements.

***OBJECTIVE 2. To determine the effects of a ketogenic diet on tumor progression.*** Primary tumor progression will be analyzed using advanced ultra-low dose fluorodeoxyglucose-positron emission tomographic (FDG-PET) imaging that will allow for simultaneous measurement of tumor metabolism and growth while also assessing normal metabolism in musculature. Tumor progression by FDG-PET may also be complemented by biochemical analysis of tumor biopsy samples as part of standard of care.

### OBJECTIVE 3. To determine the effects of nutritional ketosis on biologic and behavioral health markers.

3a. We hypothesize that sustained nutritional ketosis will decrease insulin resistance, decrease whole body and visceral fat, improve dyslipidemia, glucose management, and inflammation status.

3b. We hypothesize that nutritional ketosis will be associated with improved behavioral measures of quality of life and cancer related fatigue.

### STUDY DESIGN

We will enroll a total of 10 participants with newly diagnosed Stage 4 metastatic breast cancer (various histological cancer types) into a ketogenic diet group. This pilot cohort will receive and undergo all standard of care regiments if they elect to. The ketogenic diet arm will undergo a 6-month diet intervention, where the first three months will be a controlled feeding phase and the second three months will be free-living. In an effort to maintain a patient centric focus and monitor changes in quality of life (QOL) all patients will complete psychosocial and behavioral inventories. These inventories aim to capture a holistic view on the proposed nutritional intervention during palliative chemotherapy. Primary outcomes will be determined at baseline, 3 months, and 6 months (see section 13 – Study Calendar) while patient-centric outcomes will be assessed every six weeks.

### Eligibility Criteria

Inclusions:

- - - Age ≥18 years
    - Body mass index (BMI) ≥22 kg/m2
    - Confirmed diagnosis of metastatic or stage IV BC
    - FDG-PET avid tumors
    - Eastern Cooperative Oncology Group (ECOG) Performance status of 0-1 (0=participant has either normal activity, 1= participant has some symptoms but is nearly full ambulatory)
    - Life expectancy >6 months
    - Able and willing to follow prescribed diet intervention

Exclusion criteria:

- - - Prior chemotherapy for MBC (prior adjuvant chemotherapy permitted as long as >12 mo)
    - BMI <25 kg/m2
    - Weight change >5% within 3 months of enrollment
    - Type 1 diabetes
    - History of diabetes with retinopathy requiring treatment
    - Current use of insulin or sulfonylureads for glycemic control, or history of ketoacidosis
    - Intestinal obstruction
    - Abnormal liver function (Bilirubin >2, Albumin <3.5)
    - Abnormal renal function (GFR < 55 mL/min, creatinine >2.0, urinary albumin >1 g/day)
    - Congestive heart failure
    - Pregnant of nursing women
    - Unable to provide Informed Consent
    - Uncontrolled concurrent medical conditions that would limit compliance with study requirements

### TREATMENT PLAN

Due to the nature of this pilot trial, participants will be allowed to follow either standard of care pharmacological interventions or alternative medicine strategies. We will not exclude participants who wish to avoid pharmacological intervention. Participants will have counseling by the attending physician for additional applicable medications for any chemotherapy related side effects or toxicities. The intervention group will undergo a ketogenic diet regimen for 6 months.

**Phase 1 Controlled Feeding Period (0-3 months):** For the first 3 months, participants in the intervention group will be provided all their meals. The Ketogenic Diet will consist of <50 g carbohydrate (personalized based on level of ketones checked daily by finger stick), ~15-20% protein and ~70-75% fat. Total energy intake will be *ad libitum* to permit overweight/obese participants to restrict caloric intake to induce weight and fat loss for those who are overweight. Participants will be provided with a handheld glucometer (Precision Xtra, Abbott Nutrition) and ketone test strips in order to check the concentration of BOHB from a finger stick. Normal levels of ketones in a person consuming more than 100 grams of carbohydrate per day is <0.1 mmol/L. The goal of the diet will be to induce a state of nutritional ketosis defined as blood ketones >0.5 mmol/L. We believe that a level of ketones above 1.5 mmol/L will enhance the metabolic therapy and deliver better outcomes based on preliminary studies in women with breast cancer (Fine et al., 2012; Schmidt et al., 2011) and blood BOHB concentrations shown to inhibit histone deacetylases (Shimazu et al., 2013b).The carbohydrate level required to induce nutritional ketosis will vary from person to person and thus objective feedback provided by testing blood ketones is a novel tool we will use to personalize the diet by titrating the carbohydrate and protein intake to the participant’s individual ketosis threshold.

All the participant’s meals will be prepared in our research kitchen on the OSU campus. We have established the infrastructure for conducting controlled feeding studies for other funded research including development and implementation of 7-day rotational ketogenic menus. A wide range of whole foods will be incorporated into the menus including non- starchy vegetables, fruits (berries, olives, tomatoes, lemons/limes), meats (beef, chicken, pork, fish, lamb), nuts and seeds, oils (olive, canola, coconut), cheese, butter, cream, and eggs. Contrary to the misconception that the diet is boring or overly restrictive, it is noteworthy that even the most carbohydrate intolerant person can choose from a wide range of whole foods including berries and a wide assortment of vegetables. A 7-day ketogenic meal plan is shown in **Appendix A**. We will also utilize new ketogenic food products being developed by manufacturers (e.g., Atkins Nutritionals Inc, Quest Nutrition LLC), coconut oil which contains medium chain fatty acids that break down easily into ketones, and pre-formed ketone supplements (e.g., KetonX™) as an adjunct to the diet to enable patients to achieve a consistent level of nutritional ketosis, which again we believe will enhance the therapy.

**Phase II Free-Living Period (3 to 6 months):** For the second 3 month period, participants in the intervention group will transition into a free-living ketogenic diet, with the goal of demonstrating the feasibility of making the ketogenic diet a lifestyle. The rationale for this phase is based on the need to demonstrate the real-World implementation of a

personalized ketogenic program managed within an academic medical health center at OSU. We have developed a program that includes group format, individual sessions, and online digital content to educate people how to implement a ketogenic eating pattern into their lifestyle. The education will formally start during the last two weeks of the feeding period so that participants are ready to begin preparing appropriate ketogenic meals on their own when the feeding period ends. The core curriculum provides participants with the knowledge to consume a well-formulated, personalized, sustainable ketogenic diet. Group format sessions will be emphasized early on to teach general principles, instill a sense of control, and provide peer support. To allow for some degree of personalized learning and progression, we will incorporate flexibility in educational content by complementing group classes with one-on-one sessions to provide individualized feedback based on patient knowledge and their personal test results. Spouses or members of the family who prepare meals will be permitted to attend the sessions in order to support the patient’s dietary transition. Patients will have easy access to research staff to provide customized guidance and progression through the program.

In brief, the diet prescription will follow the same principles as the controlled feeding phase (except participants will be responsible for their own food intake) where dietary sugars and starches are restricted to a point where individuals are in nutritional ketosis. Other aspects of the diet will be prescribed to ensure safety, effectiveness, satiety, and satisfaction. This will include moderate protein intake, consumption of an appropriate quantity and quality of fat, and adequate mineral intake essential to a low carbohydrate diet. Specifically because the program is not a ‘one-size-fits-all’ diet, it has the flexibility to meet the needs of a broad range of personal preferences and to adapt to an individual’s changing needs over time. These characteristics are hypothesized to result in superior efficacy and sustainability compared to the current more casual approaches to prescriptive dieting. Additionally, daily ketone monitoring and reporting will continue during Phase II.

**Adherence and Retention:** Achieving good adherence and retention is one of the most challenging aspects of prospective diet intervention studies. Most studies of low- carbohydrate ketogenic diets have poor compliance because it is not emphasized and investigators are often ignorant of how to construct and implement them effectively. Historically we have had excellent compliance and satisfaction to a ketogenic diet in both feeding and free-living studies. Ketogenic diets should not be forced on people so we carefully explain details of the diet (including meal plans, acceptable and non-acceptable foods, etc.) so that individuals are knowledgeable about what to expect and can make an informed choice whether to enroll in a study. When all food is provided there is greater control over nutrient intakes and we ensure all the features of a well-formulated ketogenic diet are achieved. We have performed multiple ketogenic feeding experiments including an ongoing study at OSU where we have observed excellent adherence over a 10 week period as determined by daily capillary BOHB measurement and verbal communication with participants. The proposed study will be the first time we work specifically with cancer patients who are undergoing chemotherapy. We acknowledge there may be unique challenges in regards to compliance in this population of women with metastatic breast cancer due to their cancer diagnosis and/or drug-induced effects on appetite and preferences for food. This is the main reason we want to conduct a feeding study during

the first 3 months to optimize the diet composition and personalize it to their unique metabolism and preferences. This is the first time that has been done. A controlled feeding study also takes the stress off the patient to purchase and prepare different foods and meals, which requires commitment and additional time to master.

Adherence during the free-living portion of the study will be more challenging, but important from the perspective of being more real-world. We also have a successful track record of excellent compliance and satisfaction during free-living ketogenic diet intervention studies. Notably, Dr. Volek is currently involved in a 400+ clinical trial in diabetic patients who have been educated on the details of a ketogenic diet. All patients have completed 3 months of the free-living study, and the majority have completed 1 yr. They receive regular education and support via a health coach, and monitor ketones daily. Adherence was over 90% at 3 months and is over 80% at one year. Notably, the majority of patients have normalized their HbA1c (i.e., reversed their type-2 diabetes) while removing the majority of their medication (especially insulin and sulfonylureas) and losing substantial body mass (-14% at one year). Thus, ketogenic diets are sustainable and highly restorative in patients with insulin resistance when proper education and support are provided.

Due to the small sample size proposed (n=10), we will have an enhanced researcher/participant relationship to promote individualized education and counseling to facilitate nutritional ketosis and perceived benefit. We have developed extensive educational materials and resources to help participants successfully make the behavior change to eating a very low-carbohydrate diet. We anticipate a combination of group and one-on-one sessions with participants to deliver this information. Well-formulated ketogenic diets are unique when compared to other diets in that there exists an ability to receive direct feedback on participant adherence via finger prick and handheld glucometer that measures the biomarker BOHB. Enrolled participants will be in daily correspondence with research staff throughout the duration of the study to provide a personalized nutrition approach and optimize the amount of time spent in nutritional ketosis.

### Clinical assessments

*Standard of Care Clinic Visits*

Participants will be evaluated in the clinic prior to the start of chemotherapy and study intervention, as well as at monthly intervals for history, physical examination for vitals, height, and weight. Hearth Hope Index (HHI), SF-36, FACT-B, Brief Pain Inventory, and Brief Fatigue Inventory, and self-report diaries (adverse events, adherence and palatability surveys) will be completed to evaluate QOL and will be administered at baseline, monthly during the intervention and at study terminus. Serum or plasma-based biological markers will also be collected at baseline, and monthly until study terminus. FDG-PET imaging will be utilized at baseline, 3 and 6 mo to evaluate disease status and progression. Further FDG- PET imaging will be utilized to determine further tumor characteristics including their metabolic estimate of the primary tumor and metastatic sites by adding dynamic imaging after the normal iv. radiotracer injection. Body composition and bone mineral density will

be assessed at the Endocrinology and Metabolic Research Laboratory at baseline, 3 months and study terminus via the use of dual energy x-ray absorptiometry (DXA).

*Imaging Based Visits*

As PET imaging allows a non-invasive insight into the metabolism of cancers and normal tissue, it is the ideal imaging methodology. The team at the Wright Center of Innovation has extensive experience in Breast Cancer imaging and has been successfully developing advanced methodologies for dynamic imaging to enable the quantitative assessment of metabolic characteristics. Originally developed to image brown adipose tissue in young healthy subjects, the ultra-low dose methodologies are further ideal for the use in follow up examinations as the cumulative radiation burden of the series will be less than the current default standard of care dose for a single examination. In addition, the WCI team operates since more than one year the next generation digital PET/CT clinical system which is leapfrogging low dose and quantitative capabilities for human patient studies. The PET imaging will be performed for standard of care imaging at the lowest FDA approved dose of 5mCi 18F-FDG, which compares to 13mCi which us current standard of care. PET imaging is planned to be performed at at baseline, 3 and 6 months. For the proposed study overall tumor metabolic volume and change in rate kinetics will be evaluated. Dr. Knopp (co- investigator) is an internationally recognized expert in PET imaging and serves also as the Co-PI of the Imaging Core (IROC) for the NCI National Clinical Trials Network. He will oversee all imaging protocols and scans. As an example of the type of images we will capture in the proposed project, the figure below reveals visual reduction in number and intensity of the tumor burden, the ROI placement enables the assessment of the change in metabolic activity from a SUVmax 7.3 [a.u.] to 3.2 [a.u.].


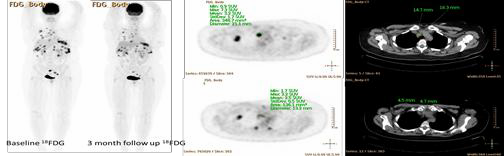


*Nutritional Research Facility Visits*

Participants in the ketogenic diet group will have additional visits 3x/week to the Nutritional Research Facility to pick up prepared foods. The research facility has a state of the art industrial kitchen with a drive up window to allow for ease of transaction. Participants will interact with the lead RD or research coordinator on the research team to discuss ketone levels, adherence, palatability and qualitative assessments of the diet.

### Adherence

Participant adherence with the study intervention will be encouraged and monitored in several ways. All participants will be provided a handheld glucometer (Precision Xtra, Abbott Nutrition) and reagent strips that measure BOHB from a small amount of blood obtained by finger stick. Although there is variability between people, BOHB levels in individuals consuming more than 100 grams of carbohydrate per day are usually ≤0.1 mmol/L. A primary goal of the initial diet intervention is for each participant to enter nutritional ketosis, which starts at 0.5 mM and extends up to approximately 3.5 mmol/L. This is a strong indication that fat is being used as one’s primary fuel, and there may be other therapeutic benefits associated with ketones in this range. Transient increases in ketones above 3.5 mmol/L may occur after exercise, but regulatory feedback systems keep ketones from elevating to dangerous levels that are seen in uncontrolled type-1 diabetics who have insufficient insulin (i.e., >10 mmol/L). Dietary assessments will be completed using free internet-based software packages (MyFitnessPal) during Phase II of the trial. This will help researchers to supervise and provide feedback on dietary choices during the free-living portion of the trial. Participants information will be kept private and only made available to IRB approved research staff. During the intervals between research- based visits participants will report their ketone levels to the research coordinator and lead RD on the team and adjustments to the diet made accordingly. Additionally, participants will be asked to take a picture of the prepared meal container both before and after ingestion to determine adherence and estimate the quantity of food consumed.

### Data and Records

Primary source documents will include forms routinely used at the James Comprehensive Breast Center, namely the Breast Patient Information Form, clinic and office notes as well as laboratory and radiology reports.

### Safety Monitoring

Adverse events will be monitored by self-reporting of signs and symptoms. Patients will maintain weekly contact with researchers and any possible ill effects will be noted and discussed with the attending physician or Research Nurse to discuss and manage any possible side effects. Patients will be counseled with regard to potential signs of treatment toxicity and should immediately contact the PI, study coordinator or treating physician in the event such a problem arises.

### Accountability

All interventional testing and feeding will be provided at no cost to the patient. Further, as a result of the time-intensive nature of the study participants in the intervention group will receive a completion stipend of $500.

### 8.0 BIOCHEMICAL AND PSYCHOSOCIAL STUDIES OVERVIEW

*Peripheral Blood Samples:*

Additional lab samples, which are not part of standard care, will be obtained at baseline and paid for as a research cost. Blood will be collected at baseline and every 4 weeks throughout the study duration until terminus.

### Insulin Resistance and Pre-Diabetes Panels

Due to the high correlation between insulin resistance, diabetes and breast cancer occurrence/prognosis, we will examine the effects of the two diets on several biomarkers including **HbA1c**, **Insulin**, **Glucose**, and **Sex hormone binding globulin (SHBG).**

### Inflammation Panel

Due to the fact that chemotherapy and cancer tumors result in increased inflammation, we will examine a panel of inflammation markers. Each of the following markers is associated with either prognosis or quality of life. The markers to be assessed will be: **TNF-α IL-6**, **IL- 8, CD-14, and CD-16.**

### Collection and Handling of Specimens

At baseline, and on a monthly basis until study terminus blood will be collected from the participant. Prior to testing all vacutainers will be labeled per study requirements. Blood will be collected into vacutainers, inverted and then stored depending on requirements for each assay.

### Tumor Biopsies

Biopsies of the tumor are often obtained as part of standard of care. We have plans to obtain specimens for archival purposes to later perform biochemical analyses as part of our interest in gaining mechanistic insight and biomarker development. However, we we are not requesting funds from Gateway to support these analyses.

### Tumor Biopsies

Optional metastatic tumor biopsies will be performed at baseline prior to start of therapy on patients who have accessible metastatic tissue that can be safely biopsied. Biopsied tissue will be tested for the Caris Target Now for molecular panel including ER, PR, Her2 neu, AR receptors, PI3-k, PTEN, EGFR, IGF-1. These will give additional information about potential mTOR resistance.

### Collection and Handling of Specimen

Patients providing consent for this optional biopsy will have tissue collected at baseline prior to the start of therapy and it will be sent to Caris Life Sciences for testing. Caris requires submission of either a tumor-containing formalin-fixed paraffin-embedded block or a total of 55 unstained, positively charged slides, each cut at 4 micron thickness. Tissue from an excisional biopsy, core needle biopsy (4-6 cores minimum, preferably using an 18 gauge needle) or fine needle aspirate (FNA) are acceptable. Sites are responsible for providing all necessary supplies for the collection and processing of the biopsy sample.

### Collection of Psychosocial and Behavioral Inventories

Psychological effects of the combinatory therapy will evaluated using several inventories including HHI, SF-36, FACT-B, Brief Pain Inventory, and the Brief Fatigue Inventory. To more directly assess the emotional and behavioral response to the diet initiation, and transition to free living diet conditions we will include the CBI-B, DSR12Q1 and FAACT. These surveys will help to determine the individual’s psychosocial response to the eating changes. Additionally, participants will be asked to maintain an event diary to determine diet palatability and any adverse event reporting. Inclusion of these surveys and diaries will provide important data on the feasibility of a ketogenic diet in breast cancer patients.

### PROCEDURES FOR PATIENT ENTRY ON STUDY

This study will be open for accrual at the James Comprehensive Breast Center at OSU. As part of the screening process, interested patients who meet initial eligibility requirements will be contacted by research staff at the Stefanie Spielman Comprehensive Breast Center and the specific requirements, risks/inconveniences, and other details of the research will be explained. Patients will have an opportunity to have all their questions and concerns about the research study addressed. The goal of this session is maximum understanding of the program requirements and its impact on their life. This process is designed to meet ethical obligations to the participant and improve retention by fostering a positive relationship between the participant and the research staff. The person obtaining informed consent will tell the patient that 1) participation is voluntary, 2) participation or non- participation will not affect their usual care and management, and 3) patient confidentiality will be maintained in the event that the results of the study are published. Patients will be provided with a consent form to review, and all questions answered. After signed informed consent has been obtained, a study identification number will be assigned to the patient for use on all data collection forms and samples.

### POTENTIAL RISKS AND MANAGEMENT

Because we will be obtaining information about a participant’s medical history, lifestyle behaviors, and measuring biomarkers that will become part of the electronic health record, there is a chance that we will uncover or discover sensitive information regarding a person’s health status. Although unlikely, this information could cause emotional distress, increase personal expense for treatment, or, if obtained by insurance companies or employers, could be used as justification to raise insurance rates or affect employability. To ensure privacy/confidentiality, information that is received from patients will be kept confidential to the extent allowed by law. Patient data will be entered into the electronic health record and hard copies will be kept in a secure filing cabinet on site for the duration of the study. We will assign all patients a code number to be used on forms, sample collection containers and other research materials. Subject codes will be employed for database management and when statistical analyses are performed. There will be a single key to the coded data kept on a password protected computer. Computer files containing names, addresses or other identifiers will be limited to authorized personnel at the site who have access to the computer data base using a password protected program. All investigators, professional medical staff, and technicians are aware of the confidentiality involved with the proper conduct of such a study. Consistent with the conduct of human research studies, the data will not be available or divulged to anyone outside of the experimental research team. The results from the study may be published, but will have no identifiers.

*Paclitaxel Treatment.* Myelosuppression, liver function test abnormalities (elevated AST, ALT, bilirubin, alkaline phosphatase), nausea, vomiting, diarrhea, mucositis, peripheral neuropathy, transient asymptomatic bradycardia, arrhythmias, hypotension, hypersensitivity/anaphylaxis reactions (dyspnea, tachycardia, rash, urticaria, hypotension, or hypertension), myalgias, arthralgias, and alopecia have been observed in patients receiving paclitaxel.

*Ketogenic Diet*. There are no significant risks associated with consuming a well-formulated ketogenic diet. For patients using medication to control blood sugar and blood pressure, there is a need to reduce these medications rather quickly at the onset of the diet to prevent low blood sugar and hypotension. In this study, we will exclude those patients who have type-2 diabetes using insulin. In our prior research we have assessed thousands of metabolic panels in patients assigned to ketogenic diets. Abnormal responses are rare, but it is expected that there will be modest changes in some metabolic parameters. These markers are expected to remain within normal limits and not pose a serious concern. For example, uric acid levels often increase during the first few weeks of a ketogenic diet and then return to or below baseline after 1-2 months. This transient increase does not exacerbate gout or have other untoward effects, since the elevation is due to competition with ketones for renal excretion, in contrast to increased intracellular synthesis of uric acid. Nutritional ketosis is associated with natriuresis (increased loss of sodium in the urine) and fluid loss. If the extra sodium excreted is not compensated for in the diet, the subsequent contracted plasma volume can manifest in side effects and adrenal stress including a hormonal response that disrupts body mineral status. Our diets contain adequate sodium and potassium to ensure mineral nutriture. The diet intervention may be challenging for participants since it will require them to limit foods they are accustomed to eating. Participants will be made aware of the general dietary requirements including lists of foods they will need to restrict (as well as foods that will be permitted) during the informational session, so they can make an educated decision to participate.

*PET Scan.* This research study involves exposure to radiation from PET scanners using the radiotracer -deoxy-2-[fluorine-18]fluoro- D-glucose (F18FDG). The total amount of radiation that each participant will receive for each scan in this study is approximately equivalent to a whole body exposure of 428 days (1.17 years) of exposure to natural background radiation. This use involves minimal risk and is routinely conducted in other research studies.

*Body Composition*. The DXA scan has a risk that is negligible, as the skin entrance dose of radiation due to the application of the exam is very small. In a whole body scan, which is the mode used in this project, the skin entrance dose of radiation per scan is ~0.04 millirem. On average in the US a person receives ~0.85 millirem per day of background radiation. For another comparison, a chest X-ray delivers ~10-20 millirems per scan. Thus the level of radiation exposure is extremely low. Since we don’t know what effect the radiation could have on an unborn baby, we will perform a urine pregnancy test before the scan for all women of child bearing age in the study.

*Blood Draws*. Blood draws by venipuncture may cause discomfort at the puncture site and the development of a slight bruise. Participants may also experience lightheadedness or fainting during the blood draw and there is a slight risk of infection. All blood draws will be taken by trained phlebotomists. The total blood volume at each testing session will be less than 50 mL, which translates into less than 200 mL over 6 months.

*Ketone Testing*. Ketone testing will be done by finger stick using a small 26G lancet. There is slight transient discomfort associated with this procedure.

This is a diet intevention study and thus no risk for toxicity exists beyond that normally present duing typical chemotherapy treatment. Nevertheless, we will record type of modification and toxicity management in detail for each patient.

The severity of adverse reactions is categorized as grade 1 to grade 5 in increasing severity. General descriptors for the toxicity grades range from none to fatal:

Grade 1 – Mild (The adverse reaction does not interfere in a significant manner with the subject’s normal functioning level. It may be an annoyance.)

Grade 2 – Moderate (The adverse reaction produces some impairment of functioning but is not hazardous to health. It is uncomfortable and/or an embarrassment)

Grade 3 – Severe (The adverse reaction produces significant impairment of funcitoning or incapacitation and is a definite hazard to the subject’s health)

Grade 4 – Adverse reactions that include or lead to either a) a life-threatening event, though acute and without permanent effect, b) prolonged inability to resume usual life pattern, or c) impairment of ability to adequately deal with future medical problems

Grade 5 – Death related to AE

Toxicity will be monitored during study visits and telephone calls using the National Cancer Institute Common Terminology Criteria for Adverse Events v4.0 (CTCAE) of the National Cancer Institute will be used (<http://evs.nci.nih.gov/ftp1/CTCAE/CTCAE_4.02_2998-09-> 15_QuickReference_5x7.pdf). Grade 3, 4 and 5 toxicities will be reported as adverse events. Patients with Grade 3-4 adverse reactions that are related to the diet will be removed from the study.

The attribution of each toxicity will be ascertained by treating physician to both standard therapy with paclitaxel as well as to diet intervention during the study. Treating physicians will manage suspected paclitaxel toxicity for dose holds and dose modifications as part of standard of care according to package insert recommendations.

### ADVERSE EVENT REPORTING

- 1. **Definition**

***Adverse event***: Any unfavorable and unintended sign (including abnormal laboratory finding), symptom, or disease temporally associated with the use of a medical treatment or procedure, regardless of whether it is considered related to the medical treatment or procedure; also an “unanticipated problem” of any nature (e.g., psychological or social harm) (designated as unrelated, definitely related, probably related, or possibly related; see below)

***Serious adverse event***: Any adverse event that is fatal or life threatening, is permanently disabling, requires inpatient hospitalization or prolongs hospitalization, or results in a congenital anomaly or birth defect

***Life-threatening event***: Any adverse event in which the subject is at immediate risk of death from the reaction as it occurs; does not include a reaction that, if it were to occur in a more serious form, might cause death

***Unexpected event***: Any adverse event that is not identified in nature, severity, or frequency in the investigator brochure, study protocol, consent form, or IND application; or the event was more serious than anticipated

## Association:

| ***Definitely Related***: An adverse event that has a timely relationship to the administ of the investigational drug/study procedure and follows a known pattern of respon which no alternative cause is present |
| --- |
| ***Probably Related***: An adverse event that has a timely relationship to the administra the investigational drug/study procedure and follows a known pattern of response, b which a potential alternative cause may be present |
| ***Possibly Related***: An adverse event that has a timely relationship to the administrat the investigational drug/study procedure, follows no known pattern of response, potential alternative cause does not exist |
| ***Unrelated***: An adverse event for which there is evidence that it is definitely relate cause other than the investigational drug/agent; in general, no timely relationship administration of the drug/procedure exists, or if so, the event does not follow a patt  response and an alternative cause is present |

The Common Terminology Criteria for Adverse Events v4.0 (CTCAE) of the National Cancer Institute will be used. The severity of adverse reactions is categorized as grade 1 to grade 5 in increasing severity. Grade 3, 4 and 5 toxicities will be reported as adverse events. General descriptors for the toxicity grades range from mild to fatal:

**Grade 1** – Mild (The adverse reaction does not interfere in a significant manner with the subject’s normal functioning level. It may be an annoyance.)

**Grade 2** – Moderate (The adverse reaction produces some impairment of functioning but is not hazardous to health. It is uncomfortable and/or an embarrassment)

**Grade 3** – Severe (The adverse reaction produces significant impairment of funcitoning or incapacitation and is a definite hazard to the subject’s health)

**Grade 4** – Adverse reactions that include or lead to either a) a life-threatening event, though acute and without permanent effect, b) prolonged inability to resume usual life pattern, or c) impairment of ability to adequately deal with future medical problems

**Grade 5** – Fatal

### Documentation

All adverse events must be documented in detail within the medical record. The patient will be observed and monitored carefully until the condition resolves, stabilizes, or its cause is identified. All adverse events, including laboratory abnormalities, will be followed up according to good medical practices. Information to be recorded includes the following:

1. Specific type of reaction.
2. Duration of reaction.
3. Severity/grade of reaction according to the NCI Common Terminology Criteria for Adverse Events v4.0 (CTCAE).
4. Suspected cause of the reaction (i.e. possibly or probably related to one of the following: study treatment, progression of disease, concurrent medications, concurrent illness, or other factors).
5. Changes made in the administration of the study drugs and other actions taken to alleviate the clinical event.
6. Patient’s response to medical interventions.

### Reporting

According to FDA regulations (21 CFR 312.32), IND safety reports shall address “any adverse experience associated with the use of a drug that is both serious and unexpected.” The IRB will be notified of any adverse event fulfilling the following criteria:

- - 1. The adverse event is **SERIOUS** (as defined above), or
    2. The adverse event is not serious, but is **UNEXPECTED** and its association with the study drug, device, or research-related procedure is either **DEFINITELY**, **PROBABLY**, or **POSSIBLY RELATED,** or **UNKNOWN** (as defined above).

Federal policy [45 CFR 46.116(b)(5)] also requires that investigators inform subjects of any important new information that might affect their willingness to continue participating in the research. When an adverse event necessitates changes to the consent/assent form(s) and/or protocol, or that notification is given to currently or previously enrolled subjects, an amendment request will be submitted in conjunction with the adverse event report. The IRB will make a determination whether any new findings, new knowledge, or adverse effects should be communicated to subjects.

In accordance with IRB guidelines, serious adverse events will be reported within 10 days of learning of the event to the Office of Research Risks Protection, Room 300, Research Foundation Building, 1960 Kenny Road, CAMPUS, 614-688-8457 telephone, 688-0366 fax, Email: [researchrisksinfo@osu.edu,](mailto:researchrisksinfo@osu.edu) using the Event Reporting Form (<http://orrp.osu.edu/irb/event/documents/EventReportingForm_v2.2.doc> ) of The Ohio State University Institutional Review Boards. If the adverse event involved the death of a subject, it will be reported immediately, usually within 72 hours. Deaths from “natural causes” or underlying disease that occur more than 30 days following completion of study interventions (i.e., events not temporally associated) need not be reported. Unexpected adverse events that are not serious but may be associated with the drug, device, or procedure (see below) should generally be reported to the IRB within 30 days of notification of the event.

In some instances, adverse events or “unanticipated problems” result in social or psychological harm rather than physical harm to subjects or others. These events should also be reported to the IRB within 30 days, unless they are considered “serious”. A letter format may be used for reporting these events instead of the Event Reporting Form, as applicable.

The IRB will review all serious adverse event reports to reevaluate the risks and benefits of the research and need for changes. All other reportable adverse events (unexpected and related or unknown) will be reviewed administratively, unless IRB review is recommended. All investigators will be notified of any action taken, usually within 30 days.

### CRITERIA FOR RESPONSE ASSESSMENT

All patients who took the intervention for any period of time will be considered evaluable. Participants with only baseline measures and who did not receive the intervention will be considered as drop-outs. RECIST v. 1.1 will be utilized to evaluate responses to therapy.

### STUDY CALENDAR

|  | **Week** | | | | | | | | |
| --- | --- | --- | --- | --- | --- | --- | --- | --- | --- |
| **Tests & observations** | **0** | **4** | **6** | **8** | **13** | **16** | **18** | **20** | **26** |
| Signed informed consent | x |  |  |  |  |  |  |  |  |
| History and Physical Exam | x |  |  |  | x |  |  |  | x |
| Height/weight | x |  |  |  | x |  |  |  | x |
| FDG-PET imaging | x |  |  |  | x |  |  |  | x |
| DXA Bone & Body Fat Quantification | x |  |  |  | x |  |  |  | x |
| Review of medications | x | x |  |  | x |  |  | x | x |
| Performance status | x | x |  | x | x | x |  | x | x |
| SF-36 | x |  |  |  | x |  |  |  | x |
| Brief Pain Inventory Questionnaire (BPI-10) | x |  | x |  | x |  | x |  | x |
| Brief Fatigue Inventory | x |  | x |  | x |  | x |  | x |
| FACT B Questionnaire | x |  | x |  | x |  | x |  | x |
| Blood tests | x |  | x |  | x |  | x |  | x |
| FFQ questionnaire/3-day food log* | x |  | x |  | x |  | x |  | x |
| Optional metastatic tumor biopsy and profiling | x |  |  |  |  |  |  |  |  |

1. ***CRITERIA FOR REMOVAL OF PATIENTS FROM PROTOCOL THERAPY***

Study patients may voluntarily withdraw at any time from the protocol. If a treating physician elects to remove a patient from the study, the Prinicpal Investigator must be notified of withdrawal from the protocol. The reasons for discontinuation of the study must be documented in the patient record and data collection forms. Patients experiencing irreversible Grade 3-4 toxicity that is clearly related to the study treatment will be removed from the protocol. Patients with documented progression of disease will be removed from the protocol.

### ETHICAL AND REGULATORY CONSIDERATIONS

This trial will be conducted in compliance with the protocol, Good Clinical Practice guidelines, and all applicable regulatory requirements.

### Institutional Review Board

The Principal Investigator will have obtained written approval to conduct the study from The Ohio State University IRB and the Clinical Scientific Review Committee of the James Cancer Hospital and Solove Research Institute. All amendments must be approved by the Institutional Review Board of The Ohio State University prior to implementation.

### Informed consent

All potential candidates for the study will be given a copy to read of the consent form for the study. The Principal Investigator and/or designee will explain all aspects of the study in lay language and answer all the candidate’s questions regarding the study. If the candidate desires to participate in the study, she will be asked to sign the Informed Consent. The study agent will not be released to a subject without a signed Informed Consent.

Elements of informed consent include explanations of 1) the purpose of the trial, 2) what the study entails, 3) alternate treatments, 4) expenses and inconveniences to be incurred, 5) discomfort and risks to the subject, 6) whether she will receive payment for participation in the study, 7) contact person to call in the event of an emergency, 8) subject rights as a result of illness or injury from trial participation, 9) her right to withdraw from the trial at any time without prejudice, 10) confidentiality of trial participation.

### Patient confidentiality

The information obtained during the conduct of this study is considered confidential and will not be released without the written permission of the subject, except as necessary for monitoring by the FDA or other regulatory agencies. All laboratory specimens will be labeled with coded identifiers in order to maintain confidentiality. Signed consent forms, data sheets, and laboratory notebooks will be kept in locked cabinets in Dr. Maryam Lustberg’s or Dr. Jeff Volek’s office and/or research laboratories.

### Publication of research findings

Publications of the research findings will present data in a format that will not reveal the identity of the participants.

### Compliance monitoring

In accordance with IRB guidelines, the study program will be reviewed by the IRB every 12 months or less. Deviations from the protocol must be documented in the medical record and reported immediately to the PI. Deviations that meet the criteria for Immediate Event Reporting (<http://orrp.osu.edu/irb/event/index.cfm>) such as those that increase risks to subjects and/or compromise scientific integrity will be reported immediately to the IRB.

### Biosafety

This project will involve the use and analysis of human cells and tissues. Specific precautions will be taken to protect laboratory personnel and support personnel form possible infective agents from these samples, with the goals of containment of biological materials, proper waste disposal, routine decontamination of equipment and surfaces, and implementation of procedures for accidents.

### Data Safety Monitoring Plan

The data and safety monitoring plan will involve the continuous evaluation of safety, data quality and data timeliness. Investigators will conduct continuous review of data and patient safety at their regular Disease Group meetings (at least monthly) and the discussion will be documented in the minutes. The Co-PIs of the trial will review toxicities and responses of the trial where applicable at these disease center meetings and determine if the risk/benefit ratio of the trial changes. Frequency and severity of adverse events will be reviewed by the Co-PIs and compared to what is known about the agent/device from other sources; including published literature, scientific meetings and discussions with the sponsors, to determine if the trial should be terminated before completion. Serious adverse events and responses will also be reviewed by the OSUCCC Data and Safety Monitoring Committee (DSMC). The Co-PIs will also submit a progress report biannually that will be reviewed by the committee per the DSMC plan. All reportable Serious Adverse Events (SAE) will also be reported to the IRB of record as per the policies of the IRB.

### STATISTICAL ANALYSES

This project will involve 10 participants in the intervention group. Given the heterogeneic nature of the pathogenesis of MBC and the response to treatment, we plan to use descriptive approaches that focus on individual responses and predictive models that attempt to identify baseline characteristics of the highest and lowest responders. If missing data is presented it will be investigated to determine the cause, missing at random or missing not at random. As discussed in the Cochrane Handbook values that are determined to be missing at random (i.e. singular missing timepoint) will be accounted for by using a weighted estimation equation*.* If handling non-random missing values (i.e. dropout or attrition), participant data will be excluded casewise for participants that fail to meet the mid-point testing or with weighted statistical models if participants exceed midpoint, respectively.

Data will be summarized by data collection time point with means, standard deviations and frequencies as appropriate. Summaries from diet assessments and ketone logs will be plotted over time to assess adherence and compliance to the ketogenic diet. Estimation of effect sizes is of the utmost importance for this feasibility study, thus we will use summary measures such as Cohen’s d to quantify changes in physiologic outcomes (e.g., change in tumor markers or inflammatory markers from baseline to 3 months), as well as psychosocial measures. Linear and nonlinear mixed models will be used as appropriate to model changes in tumor markers, comorbidity risk factors, and other measures over time, with emphasis will be on estimating effect sizes (such as slopes of change). Post hoc tests will be used to examine pairwise comparisons when significant main or interaction effects are observed. The alpha level for significance will be set at *p* ≤ 0.05.

### REFERENCES

Anders, C.K., Carey, L.A., 2009. Biology, Metastatic Patterns, and Treatment of Patients with Triple-Negative Breast Cancer. Clinical Breast Cancer 9, S73–S81. doi:10.3816/CBC.2009.s.008

Blackburn, G.L., Wang, K.A., 2007. Dietary fat reduction and breast cancer outcome: results from the Women's Intervention Nutrition Study (WINS). Am J Clin Nutr 86, s878–81.

Boden, G., Sargrad, K., Homko, C., Mozzoli, M., Stein, T.P., 2005. Effect of a Low- Carbohydrate Diet on Appetite, Blood Glucose Levels, and Insulin Resistance in Obese Patients with Type 2 Diabetes. Ann Intern Med 142, 403–411. doi:10.7326/0003-4819- 142-6-200503150-00006

Bower, J.E., 2007. Cancer-related fatigue: links with inflammation in cancer patients and survivors. Brain Behav. Immun. 21, 863–871. doi:10.1016/j.bbi.2007.03.013

Cang, S., Ma, Y., Chiao, J.-W., Liu, D., 2014. Phenethyl isothiocyanate and paclitaxel synergistically enhanced apoptosis and alpha-tubulin hyperacetylation in breast cancer cells. Exp Hematol Oncol 3, 5. doi:10.1186/2162-3619-3-5

Champ, C.E., Mishra, M.V., Showalter, T.N., Ohri, N., Dicker, A.P., Simone, N.L., 2013. Dietary recommendations during and after cancer treatment: consistently inconsistent? Nutr Cancer 65, 430–439. doi:10.1080/01635581.2013.757629

Dashti, H.M., Al-Zaid, N.S., Mathew, T.C., Al-Mousawi, M., Talib, H., Asfar, S.K., Behbahani, A.I., 2006. Long Term Effects of Ketogenic Diet in Obese Subjects with High Cholesterol Level. Mol. Cell. Biochem. 286, 1–9. doi:10.1007/s11010-005-9001-x

Feinman, R.D., Pogozelski, W.K., Astrup, A., Bernstein, R.K., Fine, E.J., Westman, E.C., Accurso, A., Frassetto, L., Gower, B.A., McFarlane, S.I., Nielsen, J.V., Krarup, T., Saslow, L., Roth, K.S., Vernon, M.C., Volek, J.S., Wilshire, G.B., Dahlqvist, A., Sundberg, R., Childers, A., Morrison, K., Manninen, A.H., Dashti, H.M., Wood, R.J., Wortman, J., Worm, N., 2015. Dietary carbohydrate restriction as the first approach in diabetes management: Critical review and evidence base. Nutrition 31, 1–13. doi:10.1016/j.nut.2014.06.011

Fine, E.J., Segal-Isaacson, C.J., Feinman, R.D., Herszkopf, S., Romano, M.C., Tomuta, N., Bontempo, A.F., Negassa, A., Sparano, J.A., 2012. Targeting insulin inhibition as a metabolic therapy in advanced cancer: a pilot safety and feasibility dietary trial in 10 patients. Nutrition 28, 1028–1035. doi:10.1016/j.nut.2012.05.001

Forsythe, C.E., Phinney, S.D., Fernandez, M.L., Quann, E.E., Wood, R.J., Bibus, D.M., Kraemer, W.J., Feinman, R.D., Volek, J.S., 2008. Comparison of low fat and low carbohydrate diets on circulating fatty acid composition and markers of inflammation. Lipids 43, 65–77. doi:10.1007/s11745-007-3132-7

Ghersi, D., Willson, M.L., Chan, M., Simes, J., 2015. Taxane‐containing regimens for

metastatic breast cancer. The Cochrane …. doi:10.1002/14651858.CD003366.pub3/full Giorgio Mustacchi, M.D.L., 2015. The role of taxanes in triple-negative breast cancer:

literature review. Drug Design, Development and Therapy 9, 4303–4318. doi:10.2147/DDDT.S86105

Gumbiner, B., Wendel, J.A., McDermott, M.P., 1996. Effects of diet composition and ketosis on glycemia during very-low-energy-diet therapy in obese patients with non-insulin- dependent diabetes mellitus. Am J Clin Nutr 63, 110–115.

Jansen, N., Walach, H., 2016. The development of tumours under a ketogenic diet in association with the novel tumour marker TKTL1: A case series in general practice. Oncology Letters.

Kozłowski, L., Zakrzewska, I., Tokajuk, P., Wojtukiewicz, M.Z., 2003. Concentration of

interleukin-6 (IL-6), interleukin-8 (IL-8) and interleukin-10 (IL-10) in blood serum of breast cancer patients. Rocz Akad Med Bialymst 48, 82–84.

Liu, K., Cang, S., Ma, Y., Chiao, J.-W., 2013. Synergistic effect of paclitaxel and epigenetic agent phenethyl isothiocyanate on growth inhibition, cell cycle arrest and apoptosis in breast cancer cells. Cancer Cell Int. 13, 10. doi:10.1186/1475-2867-13-10

Martuscello, R.T., Vedam-Mai, V., McCarthy, D.J., Schmoll, M.E., Jundi, M.A., Louviere, C.D., Griffith, B., Skinner, C.L., Suslov, O., Deleyrolle, L.P., Reynolds, B.A., 2015. A Supplemented High-Fat Low-Carbohydrate Diet for the Treatment of Glioblastoma. Clin. Cancer Res. clincanres.0916.2015. doi:10.1158/1078-0432.CCR-15-0916

McClernon, F.J., Yancy, W.S., Eberstein, J.A., Atkins, R.C., Westman, E.C., 2007. The effects of a low-carbohydrate ketogenic diet and a low-fat diet on mood, hunger, and other self- reported symptoms. Obesity (Silver Spring) 15, 182–187. doi:10.1038/oby.2007.516 Menke, A., Casagrande, S., Geiss, L., Cowie, C.C., 2015. Prevalence of and Trends in Diabetes

Among Adults in the United States, 1988-2012. JAMA 314, 1021–1029. doi:10.1001/jama.2015.10029

Newman, J.C., Verdin, E., 2014a. β-hydroxybutyrate: much more than a metabolite. Diabetes Res. Clin. Pract. 106, 173–181. doi:10.1016/j.diabres.2014.08.009

Newman, J.C., Verdin, E., 2014b. Ketone bodies as signaling metabolites. Trends in Endocrinology & Metabolism 25, 42–52. doi:10.1016/j.tem.2013.09.002

Newman, J.C., Verdin, E., 2014c. β-hydroxybutyrate: Much more than a metabolite. Diabetes Res. Clin. Pract. 106, 173–181. doi:10.1016/j.diabres.2014.08.009

Phinney, S.D., Bistrian, B.R., Evans, W.J., Gervino, E., Blackburn, G.L., 1983. The human metabolic response to chronic ketosis without caloric restriction: preservation of submaximal exercise capability with reduced carbohydrate oxidation. Metab. Clin. Exp. 32, 769–776. doi:10.1016/0026-0495(83)90106-3

Pierce, J.P., Faerber, S., Wright, F.A., Rock, C.L., Newman, V., Flatt, S.W., Kealey, S., Jones, V.E.,

Caan, B.J., Gold, E.B., Haan, M., Hollenbach, K.A., Jones, L., Marshall, J.R., Ritenbaugh, C., Stefanick, M.L., Thomson, C., Wasserman, L., Natarajan, L., Thomas, R.G., Gilpin, E.A., Women's Healthy Eating and Living (WHEL) study group, 2002. A randomized trial of the effect of a plant-based dietary pattern on additional breast cancer events and survival: the Women's Healthy Eating and Living (WHEL) Study. Control Clin Trials 23, 728–756.

Poff, A.M., Ari, C., Arnold, P., Seyfried, T.N., D'Agostino, D.P., 2014. Ketone supplementation decreases tumor cell viability and prolongs survival of mice with metastatic cancer. Int. J. Cancer 135, 1711–1720. doi:10.1002/ijc.28809

Poff, A.M., Ward, N., Seyfried, T.N., Arnold, P., D'Agostino, D.P., 2015. Non-Toxic Metabolic Management of Metastatic Cancer in VM Mice: Novel Combination of Ketogenic Diet, Ketone Supplementation, and Hyperbaric Oxygen Therapy. PLoS ONE 10, e0127407. doi:10.1371/journal.pone.0127407

Poillet-Perez, L., Despouy, G., Delage-Mourroux, R., Boyer-Guittaut, M., 2015. Interplay between ROS and autophagy in cancer cells, from tumor initiation to cancer therapy. Redox Biol 4, 184–192. doi:10.1016/j.redox.2014.12.003

Prentice, R.L., Caan, B., Chlebowski, R.T., Patterson, R., Kuller, L.H., Ockene, J.K., Margolis, K.L., Limacher, M.C., Manson, J.E., Parker, L.M., Paskett, E., Phillips, L., Robbins, J.,

Rossouw, J.E., Sarto, G.E., Shikany, J.M., Stefanick, M.L., Thomson, C.A., Van Horn, L., Vitolins, M.Z., Wactawski-Wende, J., Wallace, R.B., Wassertheil-Smoller, S., Whitlock, E.,

Yano, K., Adams-Campbell, L., Anderson, G.L., Assaf, A.R., Beresford, S.A.A., Black, H.R.,

Brunner, R.L., Brzyski, R.G., Ford, L., Gass, M., Hays, J., Heber, D., Heiss, G., Hendrix, S.L.,

Hsia, J., Hubbell, F.A., Jackson, R.D., Johnson, K.C., Kotchen, J.M., LaCroix, A.Z., Lane, D.S., Langer, R.D., Lasser, N.L., Henderson, M.M., 2006. Low-Fat Dietary Pattern and Risk of Invasive Breast Cancer: The Women's Health Initiative Randomized Controlled Dietary Modification Trial. JAMA 295, 629–642. doi:10.1001/jama.295.6.629

Santana-Davila, R., Perez, E.A., 2010. Treatment options for patients with triple-negative breast cancer. Journal of Hematology & Oncology 2010 3:1 3, 1. doi:10.1186/1756-

8722-3-42

Schmidt, M., Pfetzer, N., Schwab, M., Strauss, I., Kämmerer, U., 2011. Effects of a ketogenic diet on the quality of life in 16 patients with advanced cancer: A pilot trial. Nutrition & Metabolism 8, 54. doi:10.1186/1743-7075-8-54

Shimazu, T., Hirschey, M.D., Newman, J., He, W., Shirakawa, K., Le Moan, N., Grueter, C.A., Lim, H., Saunders, L.R., Stevens, R.D., Newgard, C.B., Farese, R.V., de Cabo, R., Ulrich, S., Akassoglou, K., Verdin, E., 2013a. Suppression of oxidative stress by β-hydroxybutyrate, an endogenous histone deacetylase inhibitor. Science 339, 211–214. doi:10.1126/science.1227166

Shimazu, T., Hirschey, M.D., Newman, J., He, W., Shirakawa, K., Le Moan, N., Grueter, C.A., Lim, H., Saunders, L.R., Stevens, R.D., Newgard, C.B., Farese, R.V., de Cabo, R., Ulrich, S., Akassoglou, K., Verdin, E., 2013b. Suppression of oxidative stress by β-hydroxybutyrate, an endogenous histone deacetylase inhibitor. Science 339, 211–214. doi:10.1126/science.1227166

Song, M.S., Salmena, L., Pandolfi, P.P., 2012. The functions and regulation of the PTEN tumour suppressor. Nature Reviews Molecular Cell Biology 13, 283–296. doi:10.1038/nrm3330

Stafford, P., Abdelwahab, M.G., Kim, D.Y., Preul, M.C., Rho, J.M., Scheck, A.C., 2010. The ketogenic diet reverses gene expression patterns and reduces reactive oxygen species levels when used as an adjuvant therapy for glioma. Nutrition & Metabolism 7, 74. doi:10.1186/1743-7075-7-74

Volek, J.S., Fernandez, M.L., Feinman, R.D., Phinney, S.D., 2008. Dietary carbohydrate restriction induces a unique metabolic state positively affecting atherogenic dyslipidemia, fatty acid partitioning, and metabolic syndrome. Progress in Lipid Research 47, 307–318. doi:10.1016/j.plipres.2008.02.003

Volek, J.S., Freidenreich, D.J., Saenz, C., Kunces, L.J., Creighton, B.C., Bartley, J.M., Davitt, P.M., Munoz, C.X., Anderson, J.M., Maresh, C.M., Lee, E.C., Schuenke, M.D., Aerni, G., Kraemer, W.J., Phinney, S.D., 2015. Metabolic characteristics of keto-adapted ultra-endurance runners. Metabolism. doi:10.1016/j.metabol.2015.10.028

# APPENDIX A

**EXAMPLE 7-DAY ROTATIONAL KETOGENIC MENU**

## Ketogenic Diet (Day 1)

### Breakfast

*Crust-less Mini Quiches Butter blend*

### Morning Snack

*Almonds, oil roasted, salted Bouillon cube, beef*

### Lunch

***Baked Salmon with dill butter and spinach***

*Salmon, Atlantic, raw*

*Butter Blend (see recipe) + Butter, salted Dill weed, dried + Onion powder*

*Black pepper + Table salt Fried Kale*

*Mushrooms, raw, white + Butter, salted*

### Afternoon Snack

*Bouillon cube, beef + Olive oil*

### Dinner

***Italian Chicken with cauliflower rice and spinach***

*Chicken thigh, meat only, raw Butter Blend*

*Basil, ground + Table salt Oregano, ground + Garlic clove*

*Cauliflower, frozen, cooked, drained Olive oil*

*Spinach, frozen, cooked, drained Butter Blend*

*Cream cheese clouds*

## Ketogenic Diet (Day 2)

### Breakfast

***Scrambled eggs with mushrooms, cheese and kielbasa***

*Egg, large, raw + Heavy whipping cream, liquid Avocado*

*Butter*

*Cheddar cheese, shredded Mushrooms, white, raw+ Canola oil Table salt*

*Bacon, pre-cooked*

### Morning Snack

*Pecans, oil-roasted, salted Bouillon cube, chicken + Olive oil*

### Lunch

***Lemon Chicken with green beans with almonds***

*Chicken thigh, meat only, raw Butter Blend + Lemon zest Table salt*

*Green beans, frozen, cooked Almonds, slivered*

*Butter Blend*

***Afternoon Snack*** *Pepperoni, beef and pork Cheddar cheese, diced Bouillon cube, chicken*

### Dinner Beef stew

*Sirloin beef, cooked + Beef broth Thyme, ground + Rosemary, dried Black pepper*

*Butter Blend + Olive oil Onions, chopped, frozen Green pepper, sweet, chopped Mushrooms, white, raw*

## Ketogenic Diet (Day 3)

### Breakfast

***Ham & Swiss omelet over spinach***

*Egg, large, raw + Olive oil Table salt*

*Ham, sliced, deli + Swiss cheese Spinach, frozen, cooked, drained Butter Blend*

### Morning Snack

*Cheddar cheese, diced Almonds, oil roasted, salted Bouillon cube, beef + Olive oil*

### Lunch

***Kielbasa with chilled mustard sauce over sautéed cabbage***

*Sausage, Polish, pork, raw*

*Dijon mustard + Mayonnaise, canola oil Cabbage, shredded + Olive oil*

*Cider vinegar + Sucralose*

### Afternoon Snack

***Celery with buffalo chicken dip***

*Blue cheese, crumbled + Mayonnaise, canola oil Blue cheese salad dressing + Hot sauce Chicken, meat only, thigh + Butter, salted Cream cheese+ Celery stalk*

### Dinner

***Spicy hamburger patty over sautéed squash and mushrooms***

*Ground beef, 73/27, raw Butter Blend*

*Table salt*

*Chili powder + Onion powder Garlic powder*

*Summer squash, julienned Mushrooms, white,*

*Butter Blend*

## Ketogenic Diet (Day 4)

### Breakfast

***Sausage, egg, and Low-Carb Pancakes with cinnamon butter***

*Low-Carb Pancakes, batter Canola oil*

*Bacon, pre-cooked*

*Egg, large, raw + Table salt Olive oil*

*Cinnamon, ground Butter Blend*

### Morning Snack Olives and cream cheese

*Cream cheese Olives, black, canned*

### Lunch

***Spaghetti squash with beef, garlic butter and parmesan***

*Spaghetti squash, cooked Garlic salt + Onion Powder Parmesan cheese, grated Ground beef, 73/27, raw Oregano, ground*

*Tomato puree Basil, ground Mascarpone parfait*

### Afternoon Snack

*Cheddar cheese, diced Bouillon cube, chicken + Olive oil*

### Dinner

***Low-Carb Cod Patties with dill sauce and cauliflower rice***

*Low-Carb Cod Patties, raw mixture Olive oil*

*Sour cream + Heavy cream Dill weed, dried*

*Cauliflower, frozen, cooked, drained Table salt*

## Ketogenic Diet (Day 5)

### Breakfast

***Low-Carb Zucchini Patties with sour cream and sausage***

*Low-Carb Zucchini Patties, raw mixture Sour cream*

*Bacon, pre-cooked Butter Blend*

### Morning Snack

*Pecans, halves, oil roasted Bouillon cube, chicken+ Olive oil*

### Lunch Taco-less salad

*Ground beef, 73/27, raw Table salt*

*Cumin, ground + Chili powder Cheddar cheese, shredded*

*Romaine lettuce, shredded + Tomatoes, red, diced Sour cream + Mayonnaise, canola oil + Salsa*

***Afternoon Snack Deviled Eggs*** *Eggs, hard boiled*

*Mayonnaise, regular + Butter blend + Dijon Mustard Black pepper*

### Dinner

***Beef with broccoli over cauliflower rice; gelatin dessert***

*Prime rib beef, raw*

*Broccoli, frozen, chop, cooked, drained Butter, salted*

*Sesame oil + Soy sauce*

*Cauliflower, frozen, cooked, drained, chopped fine Butter Blend*

*Gelatin, sugar free, prepared + Whipped cream, pressurized*

## Ketogenic Diet (Day 6)

### Breakfast

***Eggs and sausage over cauliflower hash browns***

*Eggs, large, scrambled Bacon, pre-cooked Butter blend*

*Cauliflower, frozen, cooked, drained Butter, salted + Table salt*

### Morning Snack Olives and cream cheese

*Olives, black, ripe, canned + Cream cheese Bouillon cube, chicken*

### Lunch

***Egg Salad Lettuce Wraps with a side of Fried Kale***

*Egg Salad (see recipe) Iceberg lettuce*

*Fried kale*

### Afternoon snack Guacamole with green pepper strips

*Avocado + Lemon juice Mayonnaise, regular + Garlic salt Cucumber, sliced*

*Bouillon cube, chicken*

### Dinner

***Chicken and broccoli alfredo***

*Chicken breast, meat only, boneless, skinless, raw Butter, salted*

*Broccoli, frozen, chopped, boiled, drained Alfredo Sauce*

## Ketogenic Diet (Day 7)

### Breakfast

***Breakfast Meatballs with cauliflower and tomato hash***

*Breakfast Meatballs Cauliflower, frozen, cooked, drained*

*Butter blend Tomatoes, red, diced + Table salt*

### Morning Snack

*Beef jerky*

*Bouillon cube, chicken + Olive oil*

### Lunch

***Grilled cod with citrus butter over spinach*** *Cod, Atlantic, raw + Butter Blend + Lemon juice Table salt + Orange zest*

*Lemon zest*

*Spinach, frozen, chopped cooked, drained Olive oil + Table salt*

### Afternoon Snack

*Pecans, oil roasted, salted Bouillon cube, chicken + Olive oil*

### Dinner

***Beef with peppers over zucchini and tomatoes; Cream Cheese Clouds dessert***

*Prime rib beef, cooked Green pepper, sweet*

*Tomatoes, red + Onions, chopped, frozen Summer squash (zucchini), sliced*

*Table salt Butter blend*

*Cream Cheese Clouds*
