# Supplementary material for: Feasibility and metabolic outcomes of a well-formulated ketogenic diet as an adjuvant therapeutic intervention for women with stage IV metastatic breast cancer: The Keto-CARE trial
Source: PLoS One. 2024 Jan 2;19(1):e0296523. doi: 10.1371/journal.pone.0296523 (PMC10760925; doi:10.1371/journal.pone.0296523)
Supplement: Attachment — Submitted filename: Response to Reviewers_PONE-D-23-27894.docx [file pone.0296523.s005.docx]

PLOS One [**PONE-D-23-27894**] Response to Reviewers

We want to thank the journal staff and reviewers for taking the time to carefully assess our work. This review process enhances the scientific quality and credibility of the final manuscript. Please refer to the content below for itemized responses to all the resubmission requests.

# EDITOR COMMENTS:

When submitting your revision, we need you to address these additional requirements.

1. Please ensure that your manuscript meets PLOS ONE's style requirements, including those for file naming. The PLOS ONE style templates can be found at

<https://journals.plos.org/plosone/s/file?id=wjVg/PLOSOne_formatting_sample_main_body.pdf>

<https://journals.plos.org/plosone/s/file?id=ba62/PLOSOne_formatting_sample_title_authors_affiliations.pdf>

2. We note that you have selected “Clinical Trial” as your article type. PLOS ONE requires that all clinical trials are registered in an appropriate registry (the WHO list of approved registries is at https://www.who.int/clinical-trials-registry-platform/network/primary-registries" https://www.who.int/clinical-trials-registry-platform/network/primary-registries and more information on trial registration is at http://www.icmje.org/about-icmje/faqs/clinical-trials-registration/). Please state the name of the registry and the registration number (e.g. ISRCTN or ClinicalTrials.gov) in the submission data and on the title page of your manuscript.

a) Please provide the complete date range for participant recruitment and follow-up in the methods section of your manuscript.

Additional detail is now provided under *Methods – Study Participants*.

b) If you have not yet registered your trial in an appropriate registry, we now require you to do so and will need confirmation of the trial registry number before we can pass your paper to the next stage of review. Please include in the Methods section of your paper your reasons for not registering this study before enrolment of participants started. Please confirm that all related trials are registered by stating: “The authors confirm that all ongoing and related trials for this drug/intervention are registered”. Please see http://journals.plos.org/plosone/s/submission-guidelines#loc-clinical-trials for our policies on clinical trials.

The authors confirm that all ongoing and related trials for this drug/intervention are registered. The trial was registered a priori to participant enrollment on ClinicalTrials.gov under [NCT03535701](https://www.clinicaltrials.gov/study/NCT03535701). The NCT code is listed in *Methods – Study Participants*.

3. We note that the grant information you provided in the ‘Funding Information’ and ‘Financial Disclosure’ sections do not match.

When you resubmit, please ensure that you provide the correct grant numbers for the awards you received for your study in the ‘Funding Information’ section.

4. Thank you for stating the following in the Competing Interests section:

[I have read the journal's policy and the authors of this manuscript have the following competing interests:

JSV receives royalties from book sales; is a founder and has equity in Virta Health; and is a science advisor for Simply Good Foods and Cook Keto.  MBL has received consulting fees from AstraZeneca, Biotheranostics, Novartis, Pfizer and PledPharma. The remaining authors have no relevant financial or non-financial interests to disclose.].

Please confirm that this does not alter your adherence to all PLOS ONE policies on sharing data and materials, by including the following statement: ""This does not alter our adherence to  PLOS ONE policies on sharing data and materials.” (as detailed online in our guide for authors http://journals.plos.org/plosone/s/competing-interests).  If there are restrictions on sharing of data and/or materials, please state these. Please note that we cannot proceed with consideration of your article until this information has been declared.

Please include your updated Competing Interests statement in your cover letter; we will change the online submission form on your behalf.

The statement “this does not alter our adherence to PLOS ONE policies on sharing data and materials” was included in the last paragraph of the cover letter. Furthermore, data has been uploaded to a digital repository for additional clarity (see section #5 below for more details).

5. We note that you have indicated that data from this study are available upon request. PLOS only allows data to be available upon request if there are legal or ethical restrictions on sharing data publicly. For more information on unacceptable data access restrictions, please see http://journals.plos.org/plosone/s/data-availability#loc-unacceptable-data-access-restrictions.

In your revised cover letter, please address the following prompts:

a) If there are ethical or legal restrictions on sharing a de-identified data set, please explain them in detail (e.g., data contain potentially sensitive information, data are owned by a third-party organization, etc.) and who has imposed them (e.g., an ethics committee). Please also provide contact information for a data access committee, ethics committee, or other institutional body to which data requests may be sent.

b) If there are no restrictions, please upload the minimal anonymized data set necessary to replicate your study findings as either Supporting Information files or to a stable, public repository and provide us with the relevant URLs, DOIs, or accession numbers. For a list of acceptable repositories, please see http://journals.plos.org/plosone/s/data-availability#loc-recommended-repositories.

We will update your Data Availability statement on your behalf to reflect the information you provide.

We agree with the requested editorial changes and kindly request that the data availability statement is be modified to fit journal standards. The deidentified data is now available on Dryad Digital Repository:

Buga, Alex (Forthcoming 2023). Feasibility and Metabolic Outcomes of a Well-Formulated Ketogenic Diet as an Adjuvant Therapeutic Intervention for Women with Stage IV Metastatic Breast Cancer: The Keto-CARE Trial [Dataset]. Dryad. https://doi.org/10.5061/dryad.kh18932d4

6. We note that Figure 1 and 3 in your submission contain copyrighted images. All PLOS content is published under the Creative Commons Attribution License (CC BY 4.0), which means that the manuscript, images, and Supporting Information files will be freely available online, and any third party is permitted to access, download, copy, distribute, and use these materials in any way, even commercially, with proper attribution. For more information, see our copyright guidelines: http://journals.plos.org/plosone/s/licenses-and-copyright.

We require you to either (1) present written permission from the copyright holder to publish these figures specifically under the CC BY 4.0 license, or (2) remove the figures from your submission:

A. You may seek permission from the original copyright holder of Figure 1 and  3 to publish the content specifically under the CC BY 4.0 license.

We recommend that you contact the original copyright holder with the Content Permission Form (http://journals.plos.org/plosone/s/file?id=7c09/content-permission-form.pdf) and the following text:

“I request permission for the open-access journal PLOS ONE to publish XXX under the Creative Commons Attribution License (CCAL) CC BY 4.0 (http://creativecommons.org/licenses/by/4.0/). Please be aware that this license allows unrestricted use and distribution, even commercially, by third parties. Please reply and provide explicit written permission to publish XXX under a CC BY license and complete the attached form.”

Please upload the completed Content Permission Form or other proof of granted permissions as an ""Other"" file with your submission.

In the figure caption of the copyrighted figure, please include the following text: “Reprinted from [ref] under a CC BY license, with permission from [name of publisher], original copyright [original copyright year].”

B. If you are unable to obtain permission from the original copyright holder to publish these figures under the CC BY 4.0 license or if the copyright holder’s requirements are incompatible with the CC BY 4.0 license, please either i) remove the figure or ii) supply a replacement figure that complies with the CC BY 4.0 license. Please check copyright information on all replacement figures and update the figure caption with source information. If applicable, please specify in the figure caption text when a figure is similar but not identical to the original image and is therefore for illustrative purposes only.

Figure 1 (CONSORT Diagram) and Figure 3 (Menu Template) were both created in Microsoft PowerPoint using original creative elements. For Figure 3, we depict study meal photos that were taken by our study staff. Regarding Figure 1, we are of the opinion that this work may not require CC BY 4.0 licensing due to Microsoft’s policy on creative works:

“Not all creative works are protected by copyright. There are many exceptions to and limits on copyright protection. For example, copyright only protects creative works for limited periods of time. After the period of protection expires, the copyrighted work enters the public domain. If a work is in the public domain, the work may be freely used without permission from the creator of the work.”

Please advise if the generated figures meet the PLOS submission requirements. We are glad to rework the submission as the journal staff sees suiting.

# REVIEWERS' RESPONSES TO QUESTIONS:

[Note: HTML markup is below. Please do not edit.]

Reviewer's Responses to Questions

Comments to the Author

1. Is the manuscript technically sound, and do the data support the conclusions?

The manuscript must describe a technically sound piece of scientific research with data that supports the conclusions. Experiments must have been conducted rigorously, with appropriate controls, replication, and sample sizes. The conclusions must be drawn appropriately based on the data presented.

Reviewer #1: Yes

Reviewer #2: Yes

Reviewer #3: Yes

Reviewer #4: Yes

2. Has the statistical analysis been performed appropriately and rigorously?

Reviewer #1: Yes

Reviewer #2: No

Reviewer #3: No

Reviewer #4: I Don't Know

3. Have the authors made all data underlying the findings in their manuscript fully available?

The PLOS Data policy requires authors to make all data underlying the findings described in their manuscript fully available without restriction, with rare exception (please refer to the Data Availability Statement in the manuscript PDF file). The data should be provided as part of the manuscript or its supporting information, or deposited to a public repository. For example, in addition to summary statistics, the data points behind means, medians and variance measures should be available. If there are restrictions on publicly sharing data—e.g. participant privacy or use of data from a third party—those must be specified.

Reviewer #1: Yes

Reviewer #2: Yes

Reviewer #3: No

Reviewer #4: Yes

4. Is the manuscript presented in an intelligible fashion and written in standard English?

PLOS ONE does not copyedit accepted manuscripts, so the language in submitted articles must be clear, correct, and unambiguous. Any typographical or grammatical errors should be corrected at revision, so please note any specific errors here.

Reviewer #1: Yes

Reviewer #2: Yes

Reviewer #3: Yes

Reviewer #4: Yes

5. Review Comments to the Author

Please use the space provided to explain your answers to the questions above. You may also include additional comments for the author, including concerns about dual publication, research ethics, or publication ethics. (Please upload your review as an attachment if it exceeds 20,000 characters)

*Reviewer Comments Below*

# REVIEWER COMMENTS:

## Reviewer #1:

This manuscript helps to build on a growing body of literature supporting the use of KD as an adjuvant therapy for cancer. As stated, a main strength of this project is the longevity of the intervention, as there is relatively little data beyond 3 months.

Thank you for your time and positive feedback – we appreciated your helpful comments. Please refer to your itemized changes below.

-Eligibility criteria: Were any efforts made to limit additional dietary practices (e.g., intermittent fasting or supplement use)?

We did not expect participants to come into the study adhering to a feeding regimen that could be construed as fasting (i.e., 16:8, 20:4, alternate-day fasting, water fasting). All women were encouraged during the 3-month “highly-supervised” phase to adhere to a feeding pattern that resembled breakfast-lunch-snack-dinner to limit confounding results. A reason why we did not explicitly limit participants from taking supplements (i.e., multivitamins) was because we did not anticipate supplements to interfere with diet adherence and capillary ketone monitoring.

-Adherence: It seems that the primary means of assessing adherence was daily capillary ketone measurements. Was diet composition assessed during either phase? Were 24-h recalls conducted during phone calls with dietitians? It is clear from the text as is that tracking of nutrients by participants was not required or encouraged, but I am curious to know if diet composition was analyzed by the study team.

In short, we did not perform 24h recalls or other methods of quantifying specific nutrient intakes, as this was not a major goal of the study and would have required significant effort and subject burden to accomplish in a credible manner. Self-reported assessment of dietary nutrient intake is well known to be highly biased and flawed. Indeed, it is hard to have confidence in knowing the true diet composition in many dietary intervention studies, which is a major challenge for the field. We were interested in the real-World question of whether patients with a diagnosis of MBC could adhere and adapt to a ketogenic eating pattern following principles we have previous published (1,2). To that effort, we assisted them the first 3 months by providing food. Unlike most other diet interventions, compliance to the ketogenic diet can be objectively estimated by measuring circulating ketones, which we performed.

1) <https://www.frontiersin.org/articles/10.3389/fnut.2021.618520/full?crsi=662496819&cicada_org_src=healthwebmagazine.com&cicada_org_mdm=direct>
2) <https://clinicalnutritionespen.com/article/S2405-4577(23)00032-3/fulltext>

-Protein goals: Why was the range 0.6-1.0g/kg selected for protein target?

Thank you for the astute observation. This was indeed an error related to pound to kilogram conversion. The correct protein target was “1.2-1.5 g PRO/kg of reference weight.”

## Reviewer #2:

Given the growing popularity of ketogenic diets among cancer patients, and breast cancer patients in particular, this is an important study, because it provides evidence that such a diet can safely and effectively be implemented even in advanced breast cancer patients. The results and discussion are well written and comprehensive. However, I think there are also some points in the current manuscript which can be improved or clarified.

We appreciate the encouraging comments about our trial. Our responses to your comments are inserted below.

1. Introduction, pages 3/4: Reference 14 is not enough as a reference for all the anti-tumor effects of a KD that you mention. Please cite 1-3 review articles which discuss these effects in more detail.

This is a good suggestion. We included additional review papers to support the statements in the final sentence.

2. The outcomes of interest are not explicitly mentioned in the Materials and Methods section.

Agreed – the outcomes are now stated upfront in the *Methods – Experimental Design* section.

3. Page 8, line 105: Meals were free of cost, but I did not find a statement who sponsored these meals and therefore the study. Please mention the funding sources and their role in the study design.

The grant award from the Hecht Foundation provided the funds necessary to rent out a metabolic kitchen space, purchase all the raw-food items from local grocery stores, and cook/assemble daily breakfasts, lunches, dinners, and snacks for participants throughout Phase I. We clarified this statement in text to remove ambiguity.

4. Page 9, line 128: How was a protein intake of 0.6-1 g/kg lean body mass justified?

Thank you for bringing this error to our attention and apologies for the confusion –we made a mistake during the original manuscript draft. The correct protein target should have been “1.2-1.5 g PRO/kg of reference weight.” We have successfully implemented this dietary protein range throughout all past clinical feeding trials, including the KetoCARE trial, therefore, we changed line 128 to reflect this range.

5. Page 11, line 176: At which time of the day were glucose and ketone measured? Was this specified for the participants?

Capillary ketones and glucose were always monitored in the morning, before breakfast and while fasted. We clarified the *Methods – Metabolic Measurements, Blood Collection, and Analysis* section to provide more context.

6. Page 12, Statistics: The name of the test is Shapiro-Wilk, not Wilks. Did you consider an alternative test to the t-test in case of non-normality? What were the “exploratory objectives” you wanted to address? You also mention that you did repeated-measures mixed effects ANOVA followed by post-hoc correction. It would be good to write down an equation showing how this looks like in order to help the reader comprehend which variable is predicted and which are the predictors. Also, I found not results (p-values) of this analysis in the results section.

Overall Note:

To improve the clarity of our results and per journal rules, we recently uploaded the analyzed dataset in a public data repository. We hope that this data sharing plan will assist future clinicians/researchers in replicating our planned statistical comparisons.

Other Notes:

We removed the “s” and modified the name to Shapiro-Wilk.

Non-normal data would have been reported as median [interquartile range] and analyzed for significance between timepoints using the related samples Wilcoxon Signed Rank Test.

The Bonferroni correction/adjustment for multiple comparisons was used in *Table 5* to identify pairwise differences if the main effect of time for the 1 (group) x 3 (time) ANOVA returned significant. All the variables of interest were significant different over time; therefore, the *p*-values shown in the right columns of *Table 5* under “post-hoc comparisons” header represent the pairwise differences between:

- BL and Month 3;
- BL and Month 6;
- Month 3 and Month 6.

7. Were there any women with type 2 diabetes included?

No women were diagnosed with T2D prior to enrolment in the study.

Minor comments:

Page 14, line 243: Should be Figure 5, not 4.

Page 16, line 266: Should be Figure 6, not 5.

Page 17, line 288: Should be Figure 7, not 6.

Thank you for the helpful remark - the Figure numbering system should now be chronological.

## **Reviewer #3:**

Thank you for taking the time to review our manuscript. Our responses to your feedback are inserted below.

1. Please specify how sample size was estimated.

Sample size was originally calculated based on a two-group study design comparing a ketogenic to an AICR diet. Two, small pilot studies, with no control group, guided the *a priori* sample size planning:

Fine, Eugene J, C J Segal-Isaacson, Richard D Feinman, Silvia Herszkopf, Maria C Romano, Norica Tomuta, Amanda F Bontempo, Abdissa Negassa, and Joseph A Sparano. 2012. “Targeting Insulin Inhibition as a Metabolic Therapy in Advanced Cancer: a Pilot Safety and Feasibility Dietary Trial in 10 Patients.” *Nutrition* *(Burbank, Los Angeles County, Calif.)* 28 (10): 1028–35. doi:10.1016/j.nut.2012.05.001.

Schmidt, Melanie, Nadja Pfetzer, Micheal Schwab, Ingrid Strauss, and Ulrike Kämmerer. 2011. “Effects of a Ketogenic Diet on the Quality of Life in 16 Patients with Advanced Cancer: a Pilot Trial.” *Nutrition & Metabolism* 8 (1). BioMed Central Ltd: 54. doi:10.1186/1743-7075-8-54.

Based on our prior experience with ketogenic interventions in non-cancer patients – and due to the exploratory nature of this stage IV MBC intervention – we anticipated a >15% differences in markers associated with insulin resistance between conditions (i.e., WFKD and AICR conditions). Assuming 80% power at an α-level of 0.05, we calculated that *n* = 8 participants were needed to obtain statistical significance (nQuery Advisor, Statistical Solutions, Saugus, MA). Moreover, we also anticipated that women would present heterogeneous tumor subtypes at baseline and undergo various chemotherapy treatments/drugs during the study that could plausibly interfere with adherence/feasibility. Collectively, we estimated a conservative attrition rate (20% on the high end; 10% on the low end) and enrolled ***n* = 20** women to account for all variances.

As we explained in the manuscript, we had to modify the protocol to an exploratory study with a single group (ketogenic diet) design.

2. Please identify the secondary outcome.

We provided additional information in *Methods – Experimental Design* section that clarify the primary and secondary (exploratory) outcomes.

3. How did you minimize the confounding effects of diet (adjust for carbohydrate or protein and energy intake) or BMI

We do not view diet as confounding in this study design. Diet is in fact the intervention, and compliance is measured by ketones. More specifically, we were primarily studying how changing from a habitual diet to a ketogenic eating pattern affects various outcomes. In this regard, we are not interested in quantifying specific nutrient intakes and attributing certain importance or non-importance to their presence in the diet.

4. Line 58 and 59 not true

We rephrased the highlighted statement to reflect the prior publications that built on this theme.

The KETOgenic diet in Chemotherapy to Affect REcurrence of breast cancer (Keto-CARE) trial is *among the few* exploratory safety trials designed to investigate the feasibility and metabolic effects of a WFKD in women with stage IV MBC undergoing chemotherapy.

## **Reviewer #4:**

This is an important addition to the body of work supporting the use of WFKD as adjuncts to treatment in this and other cancers.

Thank you for taking the time to provide suggestions and improve the quality of our manuscript. Our responses to your comments are inserted below.

Is it proper form to hyphenate "x-month" when it is not used as a compound adjective?

<https://emareye.com/resource-center/hyphens/#:~:text=Hyphens%20are%20often%20used%20to,two%2Dyear%2Dold%20child>.

We removed the hyphens throughout the text to reflect a correct grammatical agreement between numerals and nouns.

32. Clarify: add 'dietary' = role of dietary fat

Clarified

38. Is this true even in women with lower BMI than this cohort? (theoretical "lean mass hyper-responder" may demonstrate induced rather than congenital mixed hyperlipidemia)

Our group has published dozens of studies supporting overall positive effects of ketogenic diets on lipid and lipoprotein responses and other metabolic health markers in a wide range of populations including lean women. This was summarized in the cited review article (Hyde et al).

39. Suggest 'impact' in place of 'effects'

Modified to “impact”

47. Suggest adding 'cancer-related upregulation of phosphoinositol-3 kinase (PI3K) and mammalian target of rapamycin (mTOR) signaling'

Added the full sentence after “reducing…” to preserve the main message.

93. Hypenate 'two-arm'

Hyphenated

98. Clarify: were the women you tried to recruit as controls also offered 3 months of free personalized perpared meals and daily dietetic coaching? If not, this may have been the primary reaon that they chose the WFKD option.

Yes – the design of the study was to be maintained between WFKD and AICR groups: 3 months of personalized meals; 3 subsequent months of at-home meal preparation. There was overwhelming and explicit desire to join WFKD when women were presented with the two study arm options.

146. I think it's important to mention that there was no need to track intake given that their meals were deemed nutritionally complete by the dietetic team. And without food records, how did you determine if the meals were nutritionally adequate during Phase II?

This is a good point – we were primarily motivated to see participants sustain ketosis during Phase II rather than specifically burdening them with standardized meal reporting instruments (i.e., ASA24, DHQIII). Motivated participants were often checking in with team dietitians in the way depicted in Figure 3, so the extent of nutritional adequacy was reserved to pictorial assessment of the food that was self-reported over more robust and objective nutritional evaluation. Since the study was primarily exploratory and guided by capillary ketones, there is ample opportunity to build upon our present findings to try and capture at-home feeding habits with more precise assessment methods.

172. It is unclear what 'pane zucchini cakes' are. Can you simply state 'zucchini cakes?'

Removed the word “pane” to keep just “zucchini cakes”

233. 'Passed away' (a euphemism) doesn't belong in a research paper. Replace this with 'died' (a term more in line with medical definitions). This needs to be corrected with any mention of 'passed away' in the manuscript and supporting material.

Replaced as suggested.

237. Consider restating: 'preference for non-keto desserts as comfort food'

Modified as suggested.

243. Was “fasting” considered to be any time up until their first meal? Or was it suggested that they test within a certain time frame after awakening? Any chance that women could have skipped a day if they believed their ketones might have been impacted by food choices from the prior day? Were their self-reports of ketones/glucose verified by the team, e.g., screen shots of meter readings? Or... by a review of the meter history by a team member at clinic check-ins? My apologies if somehow I missed seeing the details.

Capillary ketones and glucose were always monitored in the morning, before breakfast and while fasted. Participants shared each value with study staff by taking a picture of the glucometer/ketometer screen. We clarified line 176 earlier to provide more context for the reader when they read line 243.

249. Consider rewording: 'One participant with hyperglycemia demonstrated a 62 mg/dL decrease in glucose (-34%); the remaining participants demonstrated a more modest but still significant decrease in glucose ()'

This is a better re-wording than the original sentence. Amended as written.

267. Body composition improved against what standard? In other words, is a decease in body fat percentage always considered to be an improvement, even in the women with lower BMI at baseline? Or... were you pooling the results rather than suggesting individual improvement? If so, please make that clearer. And perhaps add a mention that in future research, if women with a lower BMI (or more rigorous assessment of body fat percentage) were included, undesirable loss of body fat would need to be more closely monitored by the dietetic team.

This is true for women on the lower BMI scale, and making individual comparisons would not be productive if loss of body fat would, in fact, be maladaptive. We rephrased the suggested sentence with a neutral statement that we hope satisfies the reviewer’s expectation for a results section: “Mean body composition changes were mainly driven by a decrease in body fat percentage (Δ: -3.7 ± 0.5 %; *p* < 0.001).”

269. Suggest rewording: To get around the awkwardness of including a percentage as the first 'word,' perhaps: 'The overall ratio of fat mass to lean body mass lost was 3.5:1 kg:kg, with 78% of the weight loss derived from body fat mass () and 22% from lean body mass ().'

Modified as suggested.

331. Yes! Noteworthy indeed! Consider emphasizing that point here; for example, by adding an adjective, such as 'especially noteworthy' or changing this to 'of utmost importance.' The 2DIRECT trial of women with Her2 negative breast cancer presented findings in a paper comparing FMD and to standard diet: the women in the intervention were not given steroid medication. COnsidering that steroids are routinely administered as part of the SOC, perhaps add a mention of that paper? <https://www.nature.com/articles/s41467-020-16138-3>

Thank you for the sharp observation . We also cited the suggested paper, which is a good bolster to the discussion paragraph.

333. I know you understand that you can't manipulate food intake outside of force feeding. Consider changing this to: "Daily capillary BHB objectively assessed dietary adherence and that data was then used by the team to adjust meal composition ()' That also helps to highlight the importance of a diet that was responsive to changing needs of the individuals. This same approach can serve to limit unintended/undesirable weight loss, at times leading to the team's decision to modify the diet even when it means a loss of ketosis. I think this is an extremely important point, often lost in the push of some researchers that advocate any means necessary to maintain a low GKI.

Great suggestion. The readability of the paragraph is smoother and the sentence transitions to the next one better than before.

337. Careful here: it sounds like you're saying that the monitoring model was 'obviating the need for diet journaling' when in fact it was your team's constant adjustments to the plan while still providing nutritonally complete meals that obviated the need for journaling.

To prevent the reader from second guessing, we reworded the sentence to be more explicit:

Our daily capillary βHB monitoring model and continuous dietitian counseling during Phase I obviated the need for diet journaling and the burden associated with dietary recall, an effect that later confirmed the efficacy of the dietary education when participants implemented the diet on their own during Phase II.

368. A decrease in serum glucose would also result in a dampened insulin response, reducing non-pharm insulin signaling. This is important given the increase in the number of insulin receptors found on cancer cells.

Noted and agreed.

370. You reference a paper on the impact of capecitabine. A strongly recommend that you add a reference to at least one paper demonstrating that same effect on women receiving PI3Ki.

Per request we added a reference that provides more context about PI3K inhibitors.

392. Excellent! Changing to another assessment for LBM might also lead to less reluctance to include women with low BMI that are lean and metabolically healthy (other than their cancer). You may have noticed that this is more often true in women with TNBC. And in fact, it is seen here in your study with 2 of the 3 women with TNBC at the lowest BMI.

Thank you – agreed on all counts.

397. In future research, consider how to use language to present the control arm in a more positive light. For example, refering to it as a Mediterranean diet might be a draw considering the current emphasis on the perceived health benefits surrounding that term.

We have multiple ongoing trials that include the Mediterranean diet as a comparison arm to WFKD. Whereas labeling the AICR diet as “Mediterranean” would not accurately reflect the nutritional guidelines, we thoroughly agree that Mediterranean Diet terminology is more appealing than ‘low-fat diet’.

407. 'Motivated' is key here to the initial choice of a diet. Did you explore why they opted for the WFKD? If so, their input could help in the design of future studies. As you are well aware, failure to recruit adequate numbers of participants has doomed many other trials right at the start.

Personal preference, WFKD novelty, and perceived beneficence from daily registered dietitian counseling stand out as the main reasons for joining the study. Being able to recruit the target population and retention were a priori concerns that motivated us to target an *n* = 20 sample. Retrospectively, self-selection into a trial has implicit bias; however, an inadvertent finding was that women with stage IV MBC overwhelmingly prefer WFKD over a standard-of-care diet.

414. As you know, retention is extremely important for future clinical trials. Did you attempt to identify (or care to speculate on) the characteristics in the subset of women who completed 6 months?

Yes – we speculated in the discussion that glycemic variability was inversely related to the time in the study. We tried to highlight this observation to inform future clinicians about such trends. It remains generally unclear whether the retention stemmed from intrinsic motivation (i.e., the diet improved QoL by managing hyperglycemia events and/or cravings) or extrinsic motivation (i.e., oncologists encouraged women to continue the study based on adaptive responses to the diet).

417. Somewhat awkward ending here. How about, '... examining a larger cohort that includes a control group and attempts to identify key characteristics of both responders and non-responders. Future studies should also consider including a follow-up protocol that assesses long-term benefits to metabolic health, quality of life, and progression-free survival.

Thank you for the rewording suggestion. The conclusion is less ambiguous and speaks to the message of the study.

Questions:

Was MCT oil suggested? If not, why not?

MCT, or other exogenous ketones, were not included in the original proposal. Part of the reason was to evaluate the true effects of WFKD, free of exogenous supplements, on whether women with stage IV MBC both could tolerate and maintain the feeding regimen as guided by daily fasting capillary BHB. Another part of the reason was because this theme had already been evaluated in 2019 by Khodabakhshi et. al. (500mL every 2 weeks; 20% of total kcal).

Khodabakhshi A, Akbari ME, Mirzaei HR, Mehrad-Majd H, Kalamian M, Davoodi SH. Feasibility, Safety, and Beneficial Effects of MCT-Based Ketogenic Diet for Breast Cancer Treatment: A Randomized Controlled Trial Study. *Nutrition and cancer*. 2020;72(4):627-34. Epub 20190909. doi: 10.1080/01635581.2019.1650942. PubMed PMID: 31496287.

Whereas MCTs are a sound strategy to transiently increase BHB dose-dependently, the intrinsic properties of MCT oil may cause mild GI distress (at doses ≥30g/day), thereby conflating the true tolerability of the WFKD and, possibly, lead to other AEs, such as diarrhea or stomach distress according to evidence from the cycling literature.

Chapman-Lopez, T. J., & Koh, Y. (2022). The Effects of Medium-Chain Triglyceride Oil Supplementation on Endurance Performance and Substrate Utilization in Healthy Populations: A Systematic Review. *Journal of obesity & metabolic syndrome*, *31*(3), 217–229. https://doi.org/10.7570/jomes22028

Did the number of meals/snacks vary according to patient preferences?

All participants received breakfasts, lunches, snacks, and dinners in ad libitum amounts.

In your Main Takeaways sidebar, you state that 'women improved metabolic health as evidenced by decreased body fat.' I think this is somewhat problematic for reasons I've stated above.

We removed the takeaway sidebar to adhere to the journal rules, thereby satisfying your earlier observation as well.

Although you don't menition supplementation, we know from real-world experience that many people in cancer treatment do include anywhere from a few to way too many. Care to include some mention on whether or not you believe this could have some impact on ketosis (e.g., ketone salts)? or. lowering glucose (e.g., berberine)?

If the speculated literature mechanisms are true, then exogenous agents that increase ketones transiently (i.e., salts, esters, MCT) – and concomitantly decrease glucose – may favorably alter cancer treatment outcomes through pleiotropic mechanisms discussed in greater detail elsewhere:

Hyde	PN, Lustberg	MB, Miller	VJ, LaFountain	RA, Volek	JS. Pleiotropic effects of nutritional ketosis: Conceptual framework for keto-adaptation as a breast cancer therapy. *Cancer Treatment and Research Communications*; 2017. p. 32-9.

In our case, it is unclear whether adding a ketone supplement or a glucose-lowering agent, such as berberine, would have modified cancer outcomes if such supplements were prescribed daily or multiple times per day. The efficacy and mechanistic explanation of augmenting ketones while simultaneously decreasing hepatic glucose output comes primarily from SGLT2i literature, notably one article cited in this manuscript:

Blow, T., Hyde, P. N., Falcone, J. N., Neinstein, A., Vasan, N., Chitkara, R., Hurd, M. A., Sardesai, S., Lustberg, M. B., Flory, J. H., Volek, J. S., & Goncalves, M. D. (2021). Treating Alpelisib-Induced Hyperglycemia with Very Low Carbohydrate Diets and Sodium-Glucose Co-Transporter 2 Inhibitors: A Case Series. *Integrative cancer therapies*, *20*, 15347354211032283. <https://doi.org/10.1177/15347354211032283>

In brief, there is without a doubt merit in replicating the present trial with exogenous ketone agents instead of pharmaceutical grade prescribed agents. To answer this question a future study should examine the sustained effects of these supplements on tumor outcomes, QoL, and general metabolic responses, such as 24-h glycemia, insulin-sensitivity, and other comprehensive metabolic panel parameters/inflammatory markers that are relevant to tumor-suppressive effects.

Although this is outside of the scope of your study, did you compare CBC/CMP values to historical controls? It would be interesting to learn what if any differences you saw, eg Na+ levels, platelets, inflammatory markers.

As you correctly asserted, the scope of this publication was narrowed to feasibility/tolerability evaluation. Extension from this central theme would be purely speculative and warrants caution if the discussion is extended outside of this area. However, we are working on future publications that speak to the metabolic findings you mentioned. We foresee needing a review alike the one you provided us herein, and we look forward to the journal inviting you for a future critical assessment of our work.

Did I miss a list of the medications that women were taking at baseline or throughout the study? For example, statin drugs are known to raise glucose (and suppress ketones) in a subset of people. I would also like to see the amount of dex that was included for each woman. That might factor into future studies of subtypes where dex is mandatory.

The list of medications provided by oncologists is listed in **Table 1 | Participant Descriptives**. Additional information is in the supplemental files. Unfortunately, we do not have the data to answer the precise dosing strategy of dexamethasone; however, we do know that none of the participants were on statin therapies during the trial.

Feasibility in the general population cannot be assumed from this study. Have you adequately addressed that here?

We added a last sentence in the discussion to speak to this request:

“Moreover, our feasibility and adherence findings must be viewed with the understanding of our study methods and research sample, and merit attention if extended to the general population.”

Did you consider any of these references?

<https://www.frontiersin.org/articles/10.3389/fnins.2020.00390/full>

<https://link.springer.com/article/10.1007/s11060-020-03417-8>

<https://ascopubs.org/doi/abs/10.1200/JCO.2016.34.15_suppl.e23173>

These references are very good suggestions. All three studies were added to the manuscript. Thank you for taking the time to provide the links and additional background.

6. PLOS authors have the option to publish the peer review history of their article (what does this mean?). If published, this will include your full peer review and any attached files.

If you choose “no”, your identity will remain anonymous but your review may still be made public.

Do you want your identity to be public for this peer review? For information about this choice, including consent withdrawal, please see our Privacy Policy.

Reviewer #1: No

Reviewer #2: Yes: Rainer J. Klement

Reviewer #3: No

Reviewer #4: No

[NOTE: If reviewer comments were submitted as an attachment file, they will be attached to this email and accessible via the submission site. Please log into your account, locate the manuscript record, and check for the action link "View Attachments". If this link does not appear, there are no attachment files.]

While revising your submission, please upload your figure files to the Preflight Analysis and Conversion Engine (PACE) digital diagnostic tool, https://pacev2.apexcovantage.com/. PACE helps ensure that figures meet PLOS requirements. To use PACE, you must first register as a user. Registration is free. Then, login and navigate to the UPLOAD tab, where you will find detailed instructions on how to use the tool. If you encounter any issues or have any questions when using PACE, please email PLOS at figures@plos.org. Please note that Supporting Information files do not need this step.

All the figures in this manuscript (1-7) were scanned through the PACE diagnostic tool.
