# Supplementary material for: Feasibility and metabolic outcomes of a well-formulated ketogenic diet as an adjuvant therapeutic intervention for women with stage IV metastatic breast cancer: The Keto-CARE trial
Source: PLoS One. 2024 Jan 2;19(1):e0296523. doi: 10.1371/journal.pone.0296523 (PMC10760925; doi:10.1371/journal.pone.0296523)
Supplement: Attachment — Submitted filename: Response to Reviewers_PONE-D-23-27894R1.docx [file pone.0296523.s006.docx]

PLOS One [**PONE-D-23-27894R1**]

Response to Reviewers

December 2023 Revision

# ADDITIONAL EDITOR COMMENTS:

## Comment 1:

- Thank you very carefully addressing all comments provided by the reviewers. The quality of the manuscript has improved sufficiently to get published in principal, but there are two points remaining: In your response to reviewer #2 who advised to provide more statistical details you mentioned referred to the publicly available dataset. However, the link you provided is not working or does not exist, respectively (DOI 10.5061/dryad.kh18932d4).
  - Thank you again for considering our work for publication in your journal.
  - Our sincere apologies for including a faulty data repository URL – the previous DOI link was not accessible because the dataset was set to “private until publication.” Please use the **following reviewer link** to access the uploaded files:
    - <https://datadryad.org/stash/share/1Q65M9tlGx41i_K25eZF-DowV2wqa8FX6Z3yF4oVcOo>
  - This link will become publicly available **after** the paper is recommended for acceptance:
    - <https://doi.org/10.5061/dryad.kh18932d4>

## Comment 2:

- Furthermore, please specify more precisely the sentence on line 226-229 "...we analyzed the main effects of time using dependent samples t-tests..." -> the main effects of time on which variables?
  - Good remark – the statistical section was too vague. We modified the text as follows:
    - “For Phase I endpoints, we analyzed the main effects of time using paired samples t-tests (BL vs. 3 MO). When including Phase II endpoints, we used a 1 (condition) x 3 (time) repeated-measures mixed-effects analysis of variance (RM ANOVA) with Bonferroni post-hoc corrections to analyze the differences between BL and 3 MO; BL and 6 MO; and 3 MO and 6 MO. We additionally compared weekly fasting glucose/βHB mean concentrations and variability between phases using an unpaired t-test (Phase I vs. Phase II).”

We hope that added clarification to these additional comments will satisfy the editor’s expectations.
