# Supplementary material for: Feasibility and metabolic outcomes of a well-formulated ketogenic diet as an adjuvant therapeutic intervention for women with stage IV metastatic breast cancer: The Keto-CARE trial
Source: PLoS One. 2024 Jan 2;19(1):e0296523. doi: 10.1371/journal.pone.0296523 (PMC10760925; doi:10.1371/journal.pone.0296523)
Supplement: Attachment — Submitted filename: Response to Reviewers_PONE-D-23-27894R2.docx [file pone.0296523.s007.docx]

PLOS One [**PONE-D-23-27894R2**]

Response to Reviewers

December 2023 Revision

Thank you for your careful assessment of our statistical work – Please refer to our responses below.

# Comments to the Author

If the authors have adequately addressed your comments raised in a previous round of review and you feel that this manuscript is now acceptable for publication, you may indicate that here to bypass the “Comments to the Author” section, enter your conflict of interest statement in the “Confidential to Editor” section, and submit your "Accept" recommendation.

Reviewer #5: (No Response)

# Is the manuscript technically sound, and do the data support the conclusions?

The manuscript must describe a technically sound piece of scientific research with data that supports the conclusions. Experiments must have been conducted rigorously, with appropriate controls, replication, and sample sizes. The conclusions must be drawn appropriately based on the data presented.

Reviewer #5: Partly

# Has the statistical analysis been performed appropriately and rigorously?

Reviewer #5: No

# Have the authors made all data underlying the findings in their manuscript fully available?

The PLOS Data policy requires authors to make all data underlying the findings described in their manuscript fully available without restriction, with rare exception (please refer to the Data Availability Statement in the manuscript PDF file). The data should be provided as part of the manuscript or its supporting information or deposited to a public repository. For example, in addition to summary statistics, the data points behind means, medians and variance measures should be available. If there are restrictions on publicly sharing data—e.g. participant privacy or use of data from a third party—those must be specified.

Reviewer #5: Yes

# Is the manuscript presented in an intelligible fashion and written in standard English?

PLOS ONE does not copyedit accepted manuscripts, so the language in submitted articles must be clear, correct, and unambiguous. Any typographical or grammatical errors should be corrected at revision, so please note any specific errors here.

Reviewer #5: Yes

# Review Comments to the Author

Please use the space provided to explain your answers to the questions above. You may also include additional comments for the author, including concerns about dual publication, research ethics, or publication ethics. (Please upload your review as an attachment if it exceeds 20,000 characters).

Reviewer #5: This manuscript reports a single arm clinical trial investigating feasibility and Metabolic outcomes of a well-formulated Ketogenic diet as an adjuvant therapeutic intervention for women with stage IV metastatic breast cancer. This design of this clinical trial is revised from a two arms study to a single arm study due to hard to recruit controls during pandemic. I have below comments.

- Please provide statistical considerations about sample size for this study.
  - Sample size for this study was based on prior work and power analysis. Two small pilot studies in a small number of patients have been published that recommended a ketogenic diet to women with advanced BC, but the level of rigor and sophistication with the diet intervention was low.

Fine, Eugene J, C J Segal-Isaacson, Richard D Feinman, Silvia Herszkopf, Maria C Romano, Norica Tomuta, Amanda F Bontempo, Abdissa Negassa, and Joseph A Sparano. 2012. “Targeting Insulin Inhibition as a Metabolic Therapy in Advanced Cancer: a Pilot Safety and Feasibility Dietary Trial in 10 Patients.” *Nutrition* (Burbank, Los Angeles County, Calif.) 28 (10): 1028–35. doi:10.1016/j.nut.2012.05.001.

Schmidt, Melanie, Nadja Pfetzer, Micheal Schwab, Ingrid Strauss, and Ulrike Kämmerer. 2011. “Effects of a Ketogenic Diet on the Quality of Life in 16 Patients with Advanced Cancer: a Pilot Trial.” *Nutrition & Metabolism* 8 (1). BioMed Central Ltd: 54. doi:10.1186/1743-7075-8-54.

Both studies did not have a control group. The proposed project was the first controlled study of a defined well-formulated ketogenic diet in women with MBC. We planned to study **10 women** in KD and control group (20 total). Based on our vast experience with ketogenic interventions in non-cancer patients, we anticipated a >15% differences in markers associated with insulin resistance between the control (low-fat) and intervention (ketogenic diet) groups. Assuming 80% power at an α-level of 5%, we calculated that **8** participants are required to obtain statistical significance (nQuery Advisor, Statistical Solutions, Saugus, MA). We enrolled 20 subjects per group (anticipating a 20% attrition rate on the high end and 10% on the low) to account for the greater variance. We are one of the few laboratories in the world who has conducted long-term ketogenic diet studies of the type proposed and our average attrition rate is <10%. All patients who take the intervention for any period will be considered evaluable. Participants with only baseline measures and who did not receive the intervention will be considered as drop-outs.

- We have also summarized the protocol language approved a priori by the Ohio State Center for Cancer Biostatistics (originally approved for a two-arm clinical trial):

“This project will involve 10 participants per intervention group (*n* = 20 total). Given the heterogeneic nature of the pathogenesis of MBC and the response to treatment, we plan to use descriptive approaches that focus on individual responses and predictive models that attempt to identify baseline characteristics of the highest and lowest responders. If missing data is presented it will be investigated to determine the cause: missing at random or missing not at random. As discussed in the Cochrane Handbook values that are determined to be missing at random (i.e., singular missing timepoint) will be accounted for by using a weighted estimation equation proprietary to the statistical software*.* If handling non-random missing values (i.e. dropout or attrition), participant data will be excluded casewise for participants that fail to meet the mid-point testing.

Data will be summarized by collection time point with means, standard deviations and frequencies as appropriate. Summaries from diet assessments and ketone/glucose values will be plotted over time to assess adherence and compliance to the ketogenic diet. Estimation of effect sizes is of the utmost importance for this feasibility study and will focus primarily on the physiologic outcomes (e.g., change in weight, body composition, and blood markers from baseline to the end of each phase). Mixed models ANOVAs will be used as appropriate to model changes over time between BL, Phase 1, and Phase 2. Post hoc tests will be used to examine pairwise comparisons when significant main or interaction effects are observed. The alpha level for significance will be set at *p* ≤ 0.05.”

- For the data shown in the uploaded Prism file, data needs to be confirmed. For example: Data at 3 MO in “Ketones” are different from data at week 12 in “*Individual Ketone Data”. They should have been consistent.
  - Thank you for bringing up this discrepancy. I think that we were unclear upfront about how the data was obtained from each datasheet:
    - The “Ketones” datasheet contains single capillary blood values that were taken in the laboratory during in-person test visits.
    - The “Individual Ketone Data” contains the weekly averages obtained from at-home values provided by each participant.
    - In other words, we used the former sheet for BL to 3 MO Phase 1 analysis, whereas the latter for Figure 7 and Table 4 descriptives.
    - We erroneously stated in the Figure 5 caption that the values were averages when in fact they were snapshots of test visits capillary measures. We clarified this statement to increase the transparency of our results.
  - We believe that these are the main reasons for confusion and why values did not appear consistent between sheets.
- In “*Clean Individual Ketone Data”, Partial has 8 samples, 5 of them have data at 3MO, which is different from n=6 described in manuscript.
  - This is a good observation and thank you for bringing it to our attention. This error only affects the text and not the statistics. We also modified the figure 7 legend and Table 4 to reflect the correct *n*-size for ketones to satisfy your precise observation.
- In the manuscript, it reported 9 Completers and 6 Partial. But in data from “*T-test BHB Partial vs. Completers”, both Completers and Partial have same N=13.
  - We laid out the data wrongly in Prism. We reworked the unpaired t-test on 9 completers vs. 5 partials for both ketones and glucose as intended. Please refer to the new sheet that includes the correct number of participants.
  - Data is privately available at: <https://datadryad.org/stash/share/1Q65M9tlGx41i_K25eZF-DowV2wqa8FX6Z3yF4oVcOo>
- It would be helpful to have one biostatistician in the team to reanalyze the data and confirm the results.
  - To satisfy the comments, we modified the data as suggested and in line with the a priori statistical plan approved by the Ohio State Center of Cancer Biostatistics. We hope that this consideration eliminates analysis ambiguity.

7. PLOS authors have the option to publish the peer review history of their article (what does this mean?). If published, this will include your full peer review and any attached files.

If you choose “no”, your identity will remain anonymous, but your review may still be made public.

Do you want your identity to be public for this peer review? For information about this choice, including consent withdrawal, please see our Privacy Policy.

Reviewer #5: No
